# Correction: Joint Estimation of Contamination, Error and Demography for Nuclear DNA from Ancient Humans

**DOI:** 10.1371/journal.pgen.1006444

**Published:** 2016-11-08

**Authors:** Fernando Racimo, Gabriel Renaud, Montgomery Slatkin

In S2 Text, some reference numbers to equations in the second to last paragraph are misprinted and appear as question marks instead of numbers. Please view the correct S2 Text below.

## Supporting Information

S2 Text Probabilistic inference using BAM files. Explanation of methodology for inferring fragment-specific error parameters in the optional BAM mode of DICE.
(PDF)
