# Supplementary material for: Joint Estimation of Contamination, Error and Demography for Nuclear DNA from Ancient Humans
Source: PLoS Genet. 2016 Apr 6;12(4):e1005972. doi: 10.1371/journal.pgen.1005972 (PMC4822957; doi:10.1371/journal.pgen.1005972)
Supplement: S2 Table — We tested both shotgun-sequencing data and SNP capture data. We used AFR as the anchor panel and EUR as the putative contaminant panel. Samples are sorted by decreasing mean coverage. We define Convergence to be true (T) if all the parameters stably converged in a region of parameter space that does not include the upper parameter boundary. Otherwise Convergence is false (F). A line separates the two Convergence classes. SNPs = number of SNPs overlapping with anchor panel. Observations = total number of base observations analyzed. SC = SNP capture. SS = shotgun sequencing. HG = hunter-gatherer. LBK = Linear Pottery culture. MN = Middle Neolithic. LN = Late Neolithic. NEA = Neanderthal. MH = Modern Human. LogPos = Log-posterior mode. Reported Cov. = Mean read coverage reported in corresponding study. For SNP capture, this is the mean coverage of the targeted SNPs. (PDF) [file pgen.1005972.s002.pdf]

ID	Study	Group	Type	Description	Reported Cov.	SNPs	Observations	Convergence	Error	Cont. (EUR)	τ_C (AFR)	τ_A	LogPos
Altai	[4]	NEA	SS	Altai Nea.	52	9500771	495741350	T	0.13%	0.92%	0.455	2.479	-3546071.79
Loschbour	[29]	MH	SS	Loschbour	22	8733958	181642481	T	0.17%	0.03%	0.025	0.631	-5905605.85
Stuttgart	[29]	MH	SS	LBK	19	8720170	157538109	T	0.14%	0.00%	0.019	0.393	-5921740.055
I0100	[31]	MH	SC	LBK	6.727	1017124	4608980	T	0.05%	1.00%	0.025	0.382	-482622.2504
I0061	[31]	MH	SC	Karelia (HG)	5.272	729066	3189601	T	0.06%	1.69%	0.027	0.472	-393315.7875
I0104	[31]	MH	SC	Corded Ware (LN)	4.184	912245	2714837	T	0.03%	14.75%	0.027	0.685	-416006.3196
I0406	[31]	MH	SC	Spain (MN)	3.947	545379	3204204	T	0.08%	0.98%	0.025	0.387	-340329.3866
I0014	[31]	MH	SC	Motala (HG)	2.709	497524	2164912	T	0.05%	3.45%	0.033	0.542	-293020.4266
Kennewick	[45]	MH	SS	Kennewick	1	5725599	9648018	F	0.53%	45.42%	0.031	4.999	-1800155.257
MA-1	[46]	MH	SS	Mal'ta	1	6969896	13578653	F	0.06%	43.76%	0.04	5	-2133722.285
I0111	[31]	MH	SC	Bell Beaker (LN)	0.731	300636	456149	F	0.00%	50.00%	0.068	4.999	-109596.2711
I0013	[31]	MH	SC	Motala (HG)	0.657	349019	788739	F	0.01%	39.06%	0.051	4.965	-161692.482
Mezmaiskaya	[4]	NEA	SS	Mezmaiskaya Nea.	0.48	4896677	6811727	F	0.30%	5.57%	0.425	4.984	-883632.4637
I0439	[31]	MH	SC	Yamnaya	0.26	176088	194152	F	0.00%	50.00%	0.086	3.731	-50398.43106
I0060	[31]	MH	SC	Bell Beaker (LN)	0.105	67741	73195	F	0.00%	49.97%	0.113	3.685	-20210.34403
I0804	[31]	MH	SC	Unetice	0.054	34069	35522	F	0.00%	50.00%	0.08	4.636	-9780.085474
