# Supplementary material for: Joint Estimation of Contamination, Error and Demography for Nuclear DNA from Ancient Humans
Source: PLoS Genet. 2016 Apr 6;12(4):e1005972. doi: 10.1371/journal.pgen.1005972 (PMC4822957; doi:10.1371/journal.pgen.1005972)
Supplement: S3 Table — We used different 1000G populations as candidate contaminants. AFR were the anchor population in all cases, so the modern human drift is with respect to Africans. Values in parentheses are 95% posterior quantiles. Except when using AFR as the contaminant, the Neanderthal drift parameter gets stuck at the upper boundary (5 drift units) of parameter space. (PDF) [file pgen.1005972.s003.pdf]

Conta-minant panel	An-chor panel	Error rate	Contamination rate	Modern human drift	Neanderthal drift	Log-posterior mode
EUR	AFR	0.295%<br>(0.284% − 0.306%)	5.568%<br>(5.472% − 5.673%)	0.425<br>(0.423 − 0.429)	4.984<br>(4.95 − 5)	-883632.4637
AMR	AFR	0.316%<br>(0.3% − 0.322%)	5.333%<br>(5.261% − 5.48%)	0.426<br>(0.422 − 0.428)	4.994<br>(4.952 − 4.999)	-884312.5366
SAS	AFR	0.328%<br>(0.317% − 0.341%)	5.203%<br>(5.097% − 5.313%)	0.426<br>(0.422 − 0.428)	4.996<br>(4.946 − 4.999)	-884684.3521
EAS	AFR	0.393%<br>(0.379% − 0.402%)	4.53%<br>(4.48% − 4.684%)	0.423<br>(0.421 − 0.426)	4.99<br>(4.887 − 4.999)	-885493.7081
AFR	AFR	0.515%<br>(0.5% − 0.525%)	0.007%<br>(0.002% − 0.126%)	0.406<br>(0.403 − 0.409)	1.756<br>(1.701 − 1774)	-889165.6704
