# Supplementary material for: Joint Estimation of Contamination, Error and Demography for Nuclear DNA from Ancient Humans
Source: PLoS Genet. 2016 Apr 6;12(4):e1005972. doi: 10.1371/journal.pgen.1005972 (PMC4822957; doi:10.1371/journal.pgen.1005972)
Supplement: S4 Table — We used different 1000G populations as candidate contaminants. In all cases, Africans were the unadmixed anchor population and Europeans were the admixed anchor population. The ancestral human drift refers to the drift in the modern human branch before the split of Europeans and Africans. The post-split European-specific and African-specific drifts were estimated separately without the archaic genome (τAfr = 0.009, τEur = 0.255). In all cases, the Neanderthal drift parameter gets stuck at the upper boundary (5 drift units) of parameter space. (PDF) [file pgen.1005972.s004.pdf]

Contaminant panel	Unadmixed anchor panel	Admixed anchor panel	Error rate	Contamination rate	Ancestral human drift	Neanderthal drift	Admixture rate	Log-posterior mode
AFR	AFR	EUR	0.517% (0.502% − 0.526%)	4.663% (4.564% − 4.787%)	0.428 (0.426 − 0.432)	4.999 (4.989 − 5)	1.609% (1.585% − 1.63%)	-1025944.516
EAS	AFR	EUR	0.71% (0.697% − 0.721%)	2.471% (2.403% − 2.564%)	0.415 (0.412 − 0.418)	4.997 (4.985 − 5)	1.486% (1.462% − 1.508%)	-1028456.347
AMR	AFR	EUR	0.727% (0.71% − 0.733%)	2.288% (2.208% − 2.361%)	0.414 (0.412 − 0.417)	4.999 (4.985 − 5)	1.482% (1.459% − 1.501%)	-1028866.312
SAS	AFR	EUR	0.724% (0.709% − 0.732%)	2.315% (2.219% − 2.375%)	0.414 (0.412 − 0.418)	4.998 (4.984 − 5)	1.479% (1.458% − 1.5%)	-1028823.568
EUR	AFR	EUR	0.761% (0.745% − 0.77%)	1.875% (1.784% − 1.928%)	0.413 (0.41 − 0.415)	4.998 (4.984 − 2.5)	1.463% (1.457% − 1.495%)	-1029429.156
