# Supplementary figures and images for: Joint Estimation of Contamination, Error and Demography for Nuclear DNA from Ancient Humans
Source: PLoS Genet. 2016 Apr 6;12(4):e1005972. doi: 10.1371/journal.pgen.1005972 (PMC4822957; doi:10.1371/journal.pgen.1005972)

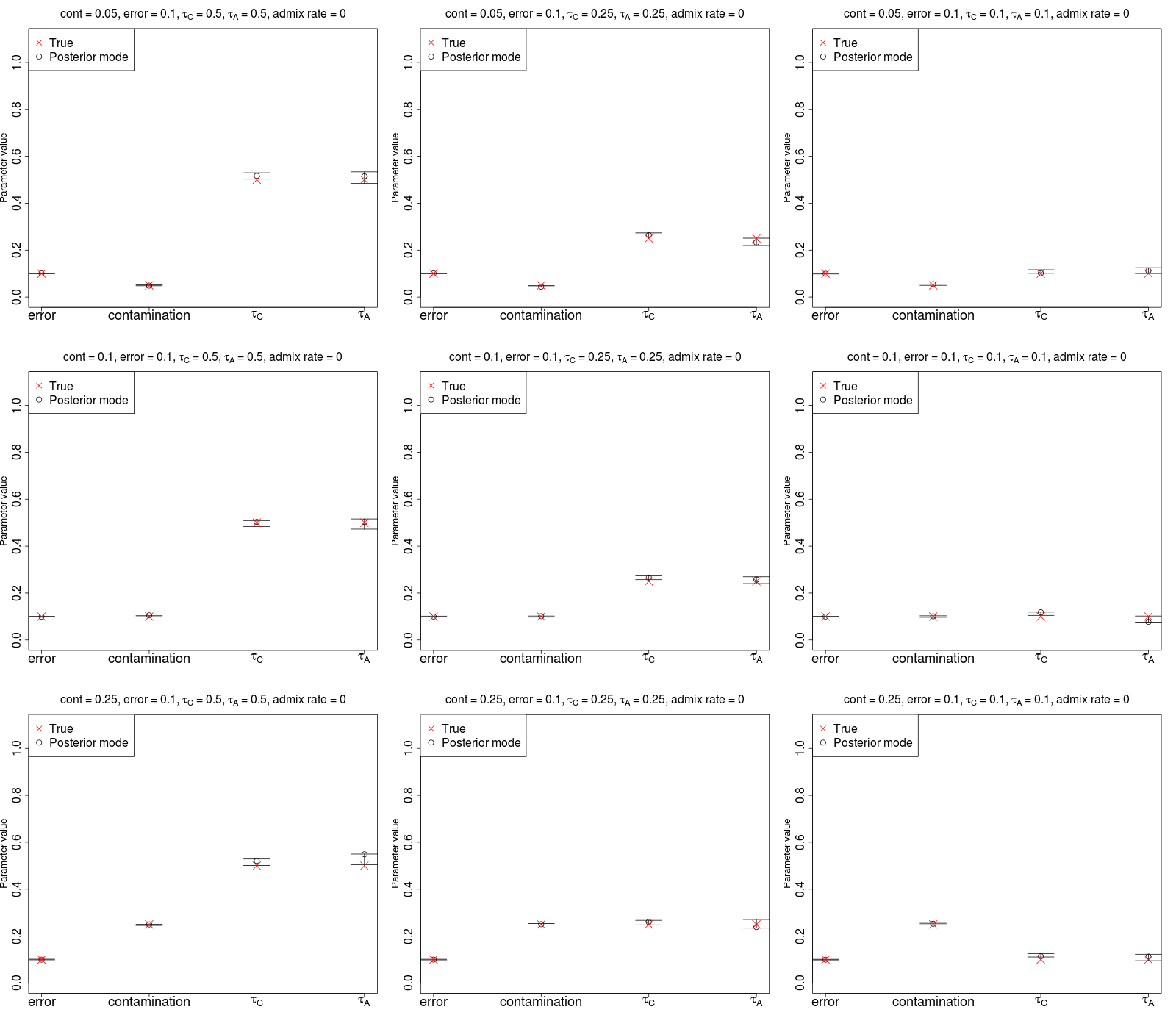

Supplement: S1 Fig — Error bars represent 95% posterior intervals. (TIFF) [file pgen.1005972.s005.tiff]

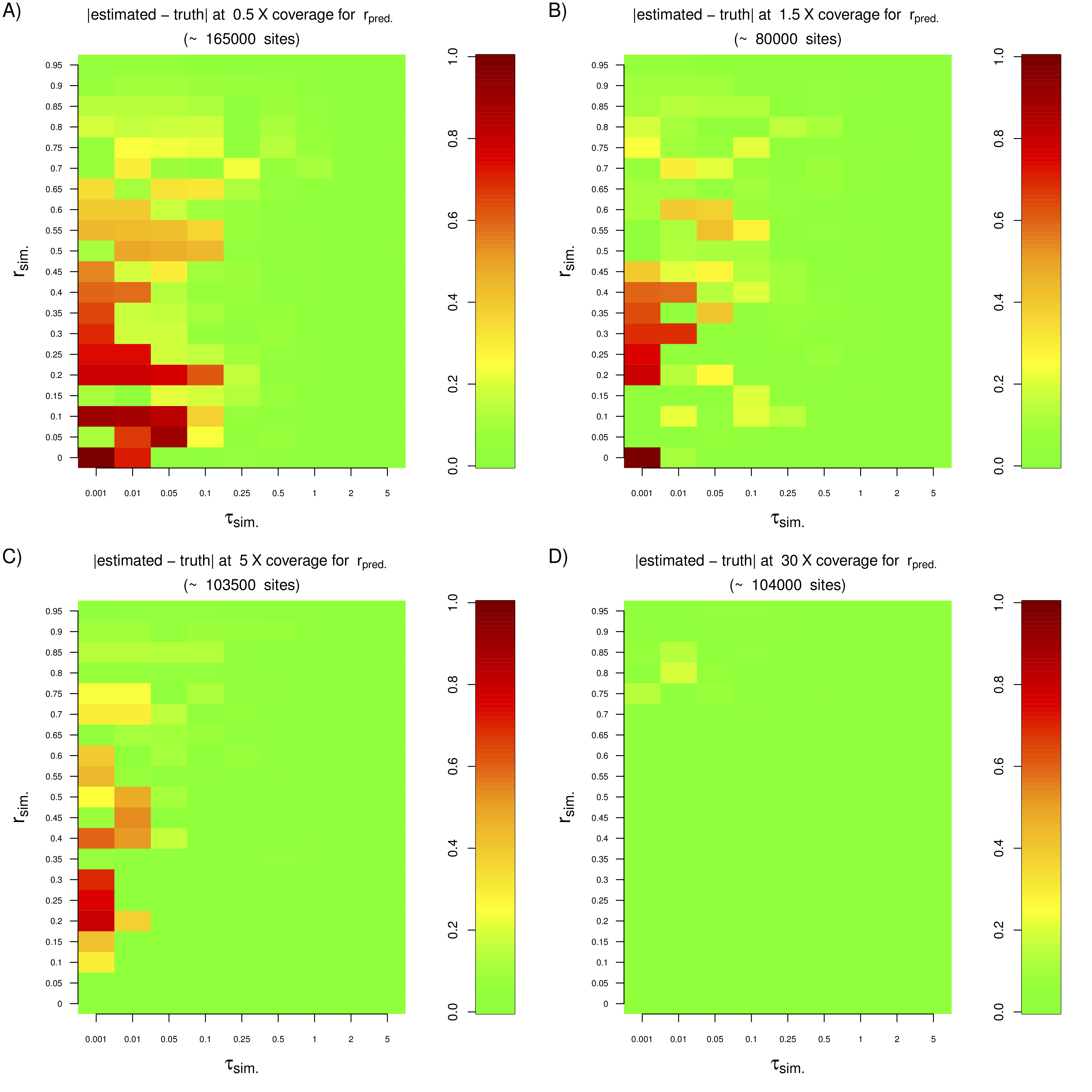

Supplement: S2 Fig — In all simulations, the anchor drift was set to be equal to the ancient sample drift. A) 0.5X coverage (800,000 simulations). B) 1.5X coverage (200,000 simulations). C) 5X coverage (200,000 simulations). D) 30X coverage (200,000 simulations). The number of sites with coverage > 0 is denoted at the top of each panel. (TIFF) [file pgen.1005972.s006.tiff]

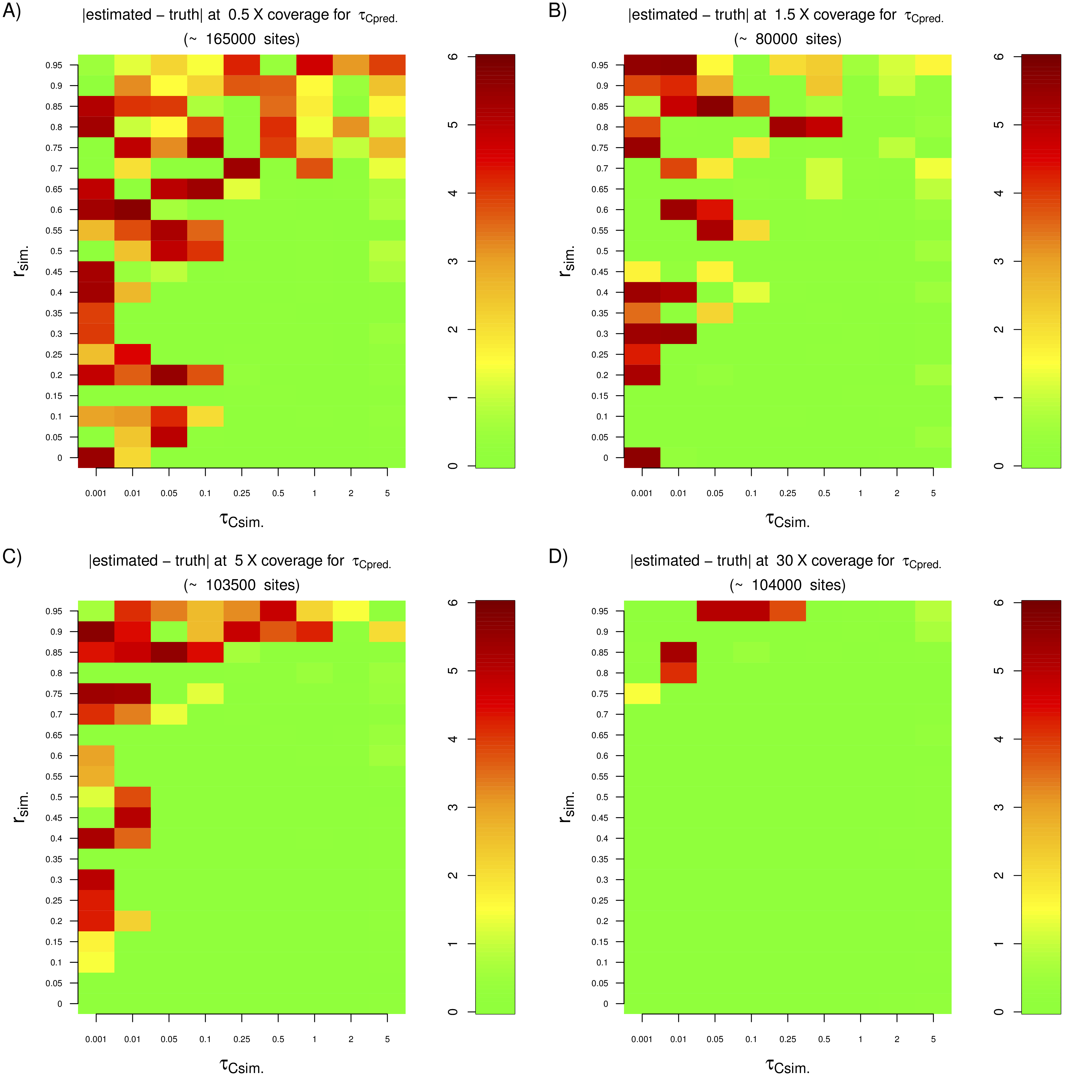

Supplement: S3 Fig — In all simulations, the anchor drift was set to be equal to the ancient sample drift. A) 0.5X coverage (800,000 simulations). B) 1.5X coverage (200,000 simulations). C) 5X coverage (200,000 simulations). D) 30X coverage (200,000 simulations). The number of sites with coverage > 0 is denoted at the top of each panel. (TIFF) [file pgen.1005972.s007.tiff]

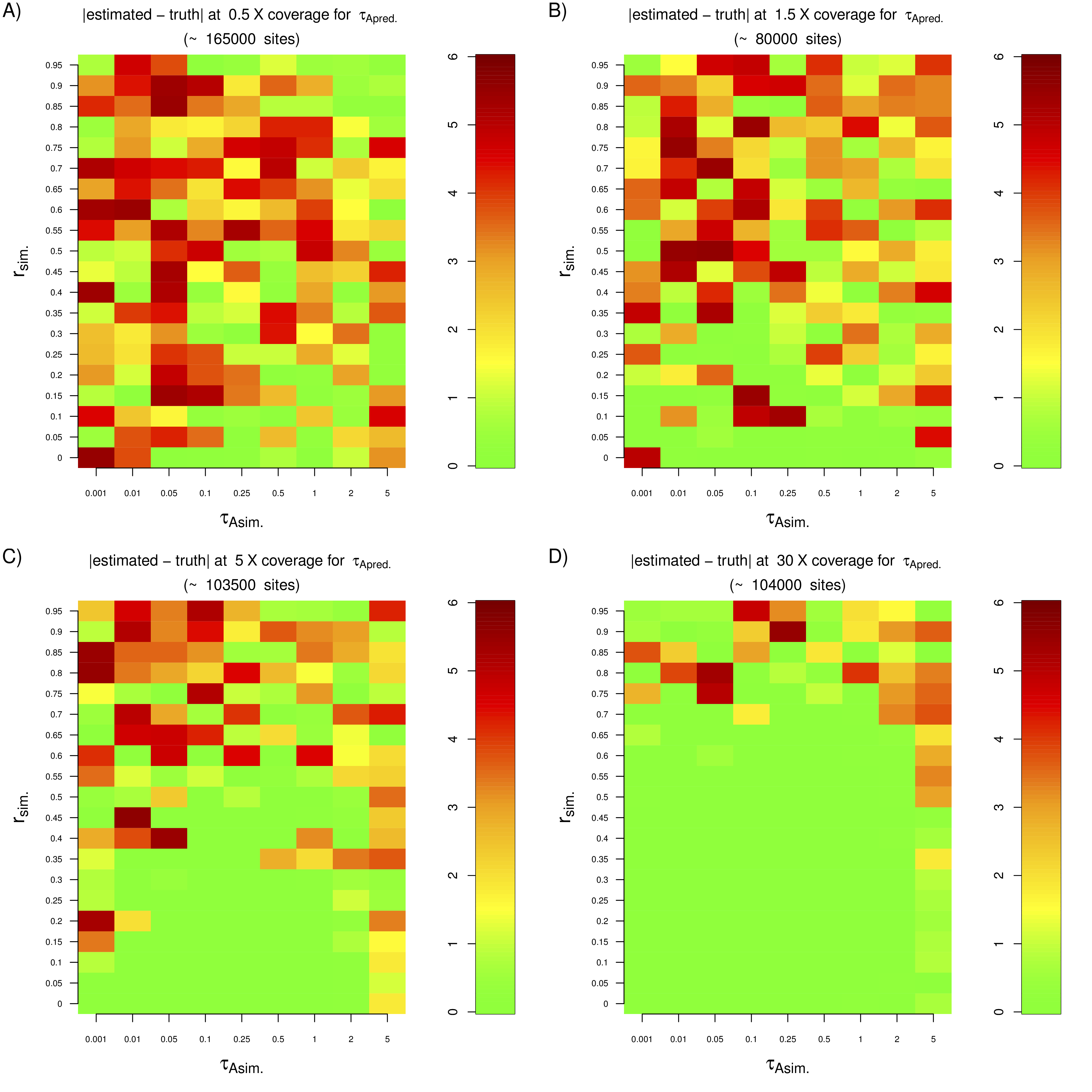

Supplement: S4 Fig — In all simulations, the anchor drift was set to be equal to the ancient sample drift. A) 0.5X coverage (800,000 simulations). B) 1.5X coverage (200,000 simulations). C) 5X coverage (200,000 simulations). D) 30X coverage (200,000 simulations). The number of sites with coverage > 0 is denoted at the top of each panel. (TIFF) [file pgen.1005972.s008.tiff]

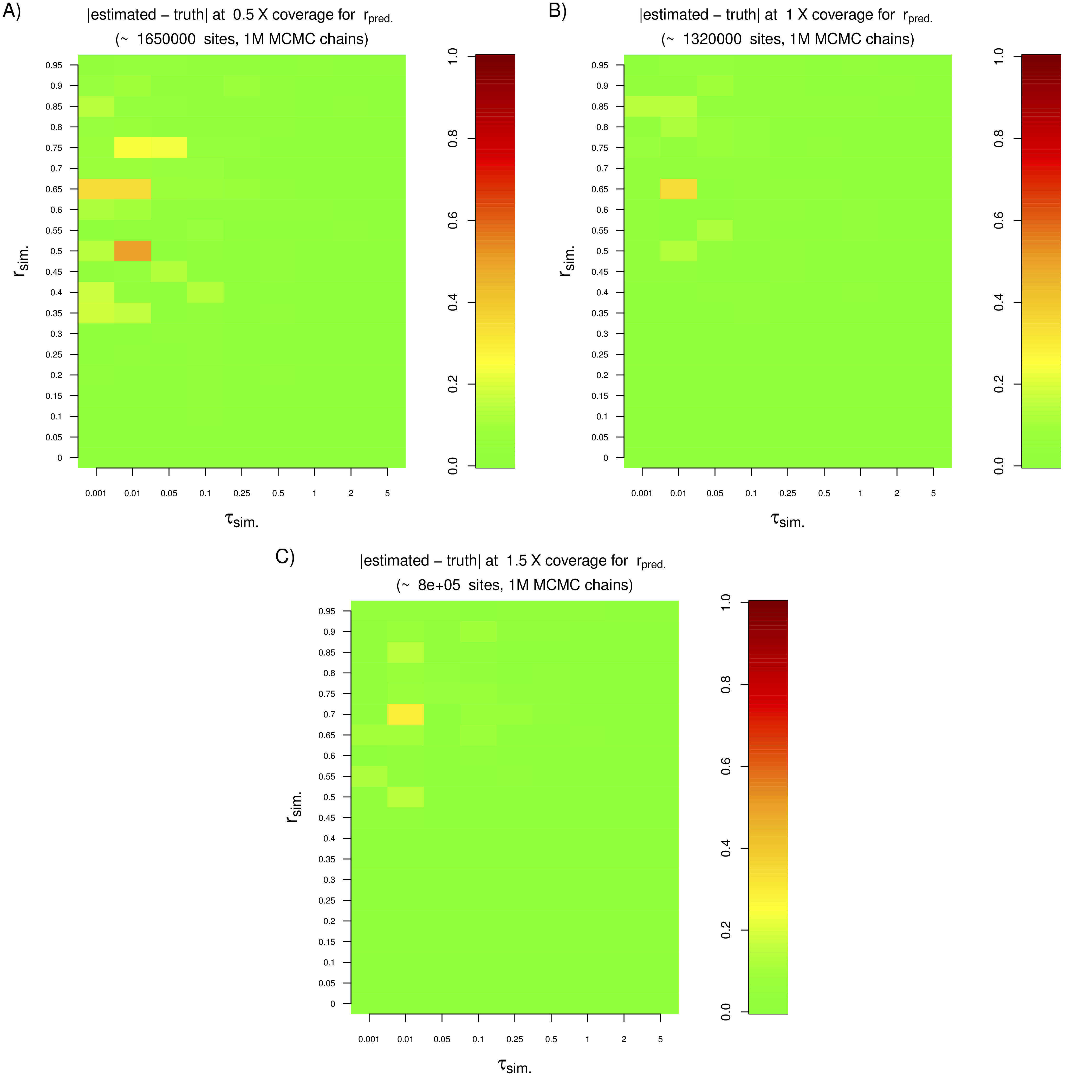

Supplement: S5 Fig — Here, we used a large number of sites and run the MCMC chain for 1 million steps. In all simulations, the anchor drift was set to be equal to the ancient sample drift. A) 0.5X coverage (800,000 simulations). B) 1X coverage (400,000 simulations). C) 1.5X coverage (200,000 simulations). The number of sites with coverage > 0 is denoted at the top of each panel. (TIFF) [file pgen.1005972.s009.tiff]

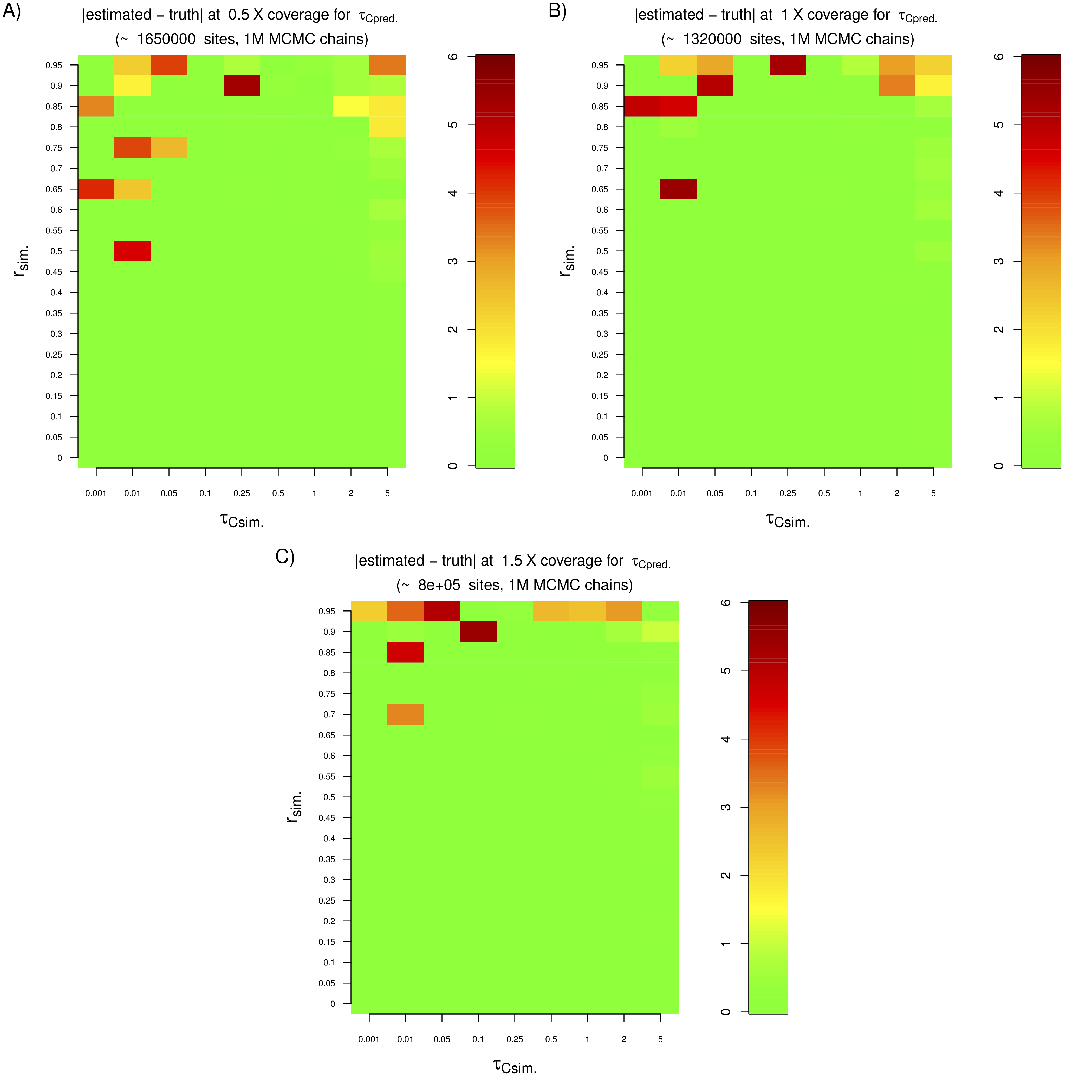

Supplement: S6 Fig — Here, we used a large number of sites and run the MCMC chain for 1 million steps. In all simulations, the anchor drift was set to be equal to the ancient sample drift. A) 0.5X coverage (800,000 simulations). B) 1X coverage (400,000 simulations). C) 1.5X coverage (200,000 simulations). The number of sites with coverage > 0 is denoted at the top of each panel. (TIFF) [file pgen.1005972.s010.tiff]

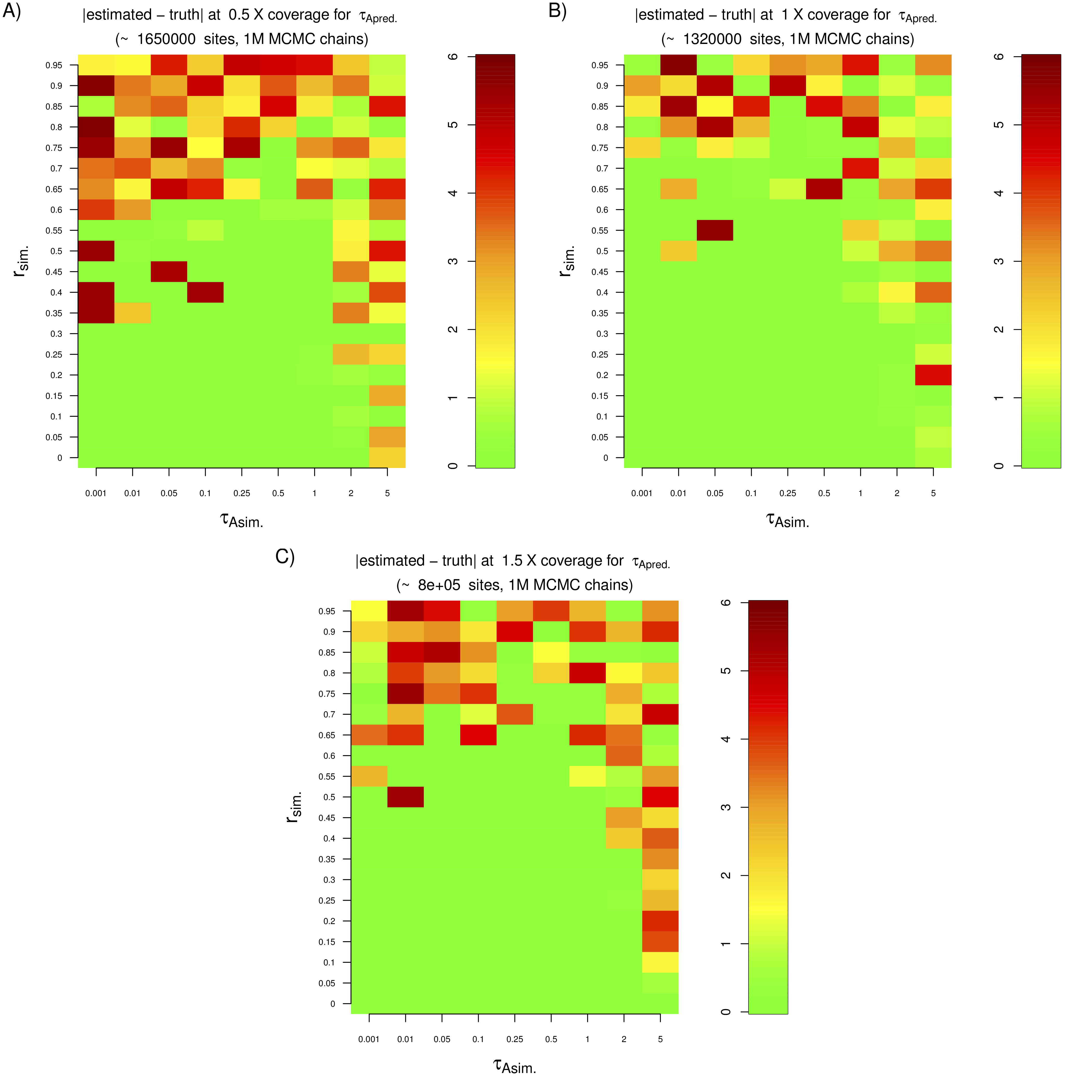

Supplement: S7 Fig — Here, we used a large number of sites and run the MCMC chain for 1 million steps. In all simulations, the anchor drift was set to be equal to the ancient sample drift. A) 0.5X coverage (800,000 simulations). B) 1X coverage (400,000 simulations). C) 1.5X coverage (200,000 simulations). The number of sites with coverage > 0 is denoted at the top of each panel. (TIFF) [file pgen.1005972.s011.tiff]

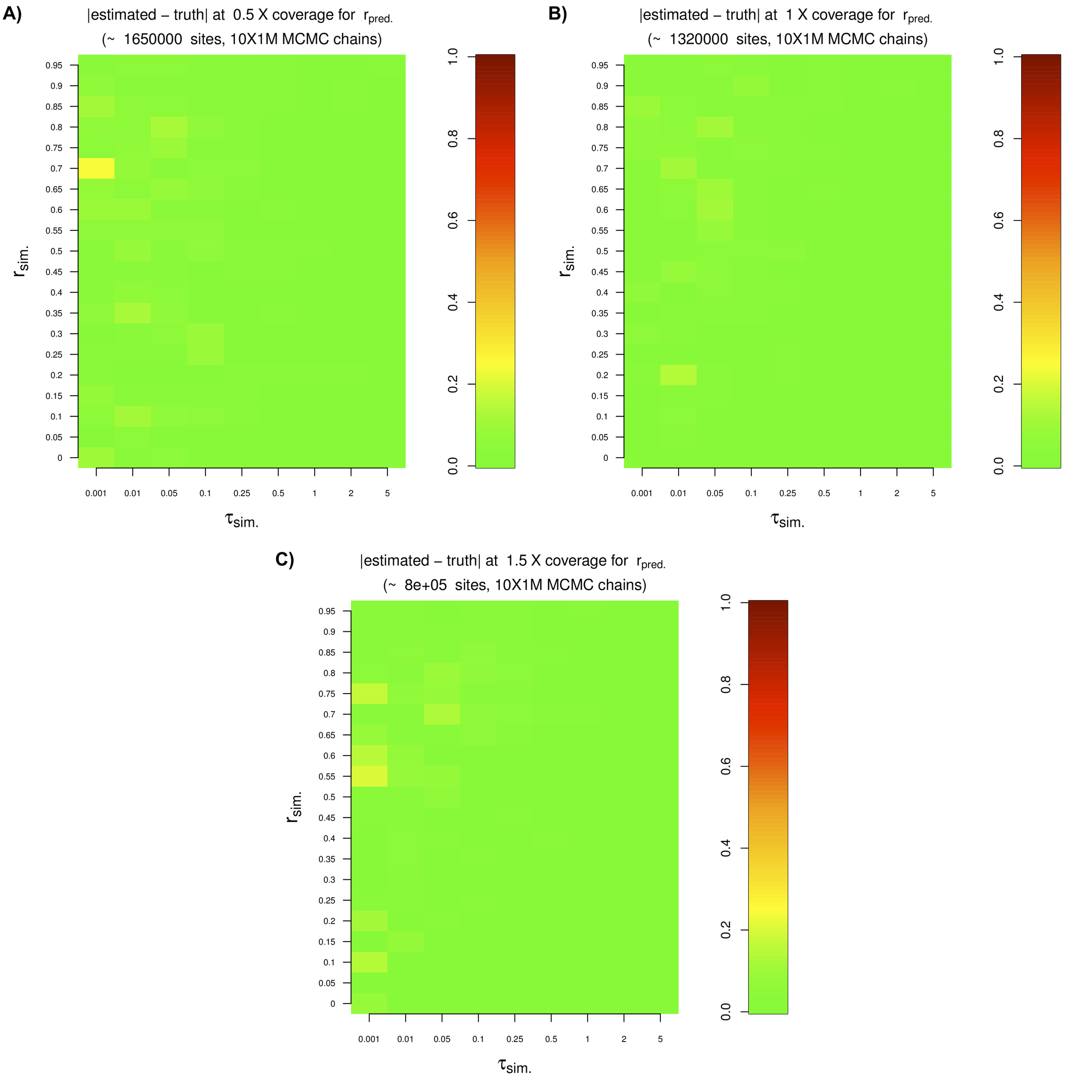

Supplement: S8 Fig — We used a large number of sites and run 10 MCMC chains for 1 million steps each. To ensure convergence, we then selected the chain with the highest posterior probability, and here show estimates from that chain. In all simulations, the anchor drift was set to be equal to the ancient sample drift. A) 0.5X coverage (800,000 simulations). B) 1X coverage (400,000 simulations). C) 1.5X coverage (200,000 simulations). The number of sites with coverage > 0 is denoted at the top of each panel. (TIFF) [file pgen.1005972.s012.tiff]

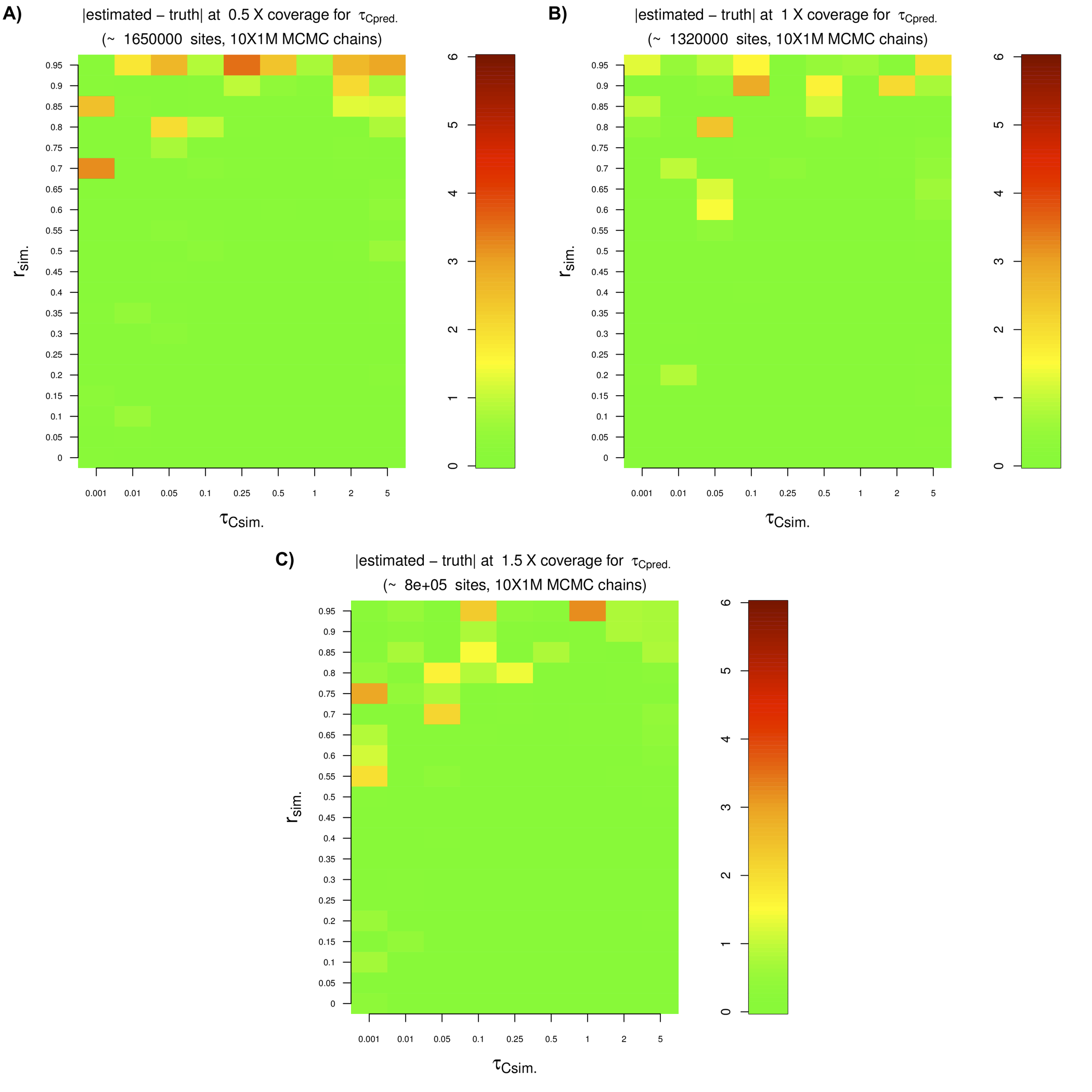

Supplement: S9 Fig — We used a large number of sites and run 10 MCMC chains for 1 million steps each. To ensure convergence, we then selected the chain with the highest posterior probability, and here show estimates from that chain. In all simulations, the anchor drift was set to be equal to the ancient sample drift. A) 0.5X coverage (800,000 simulations). B) 1X coverage (400,000 simulations). C) 1.5X coverage (200,000 simulations). The number of sites with coverage > 0 is denoted at the top of each panel. (TIFF) [file pgen.1005972.s013.tiff]

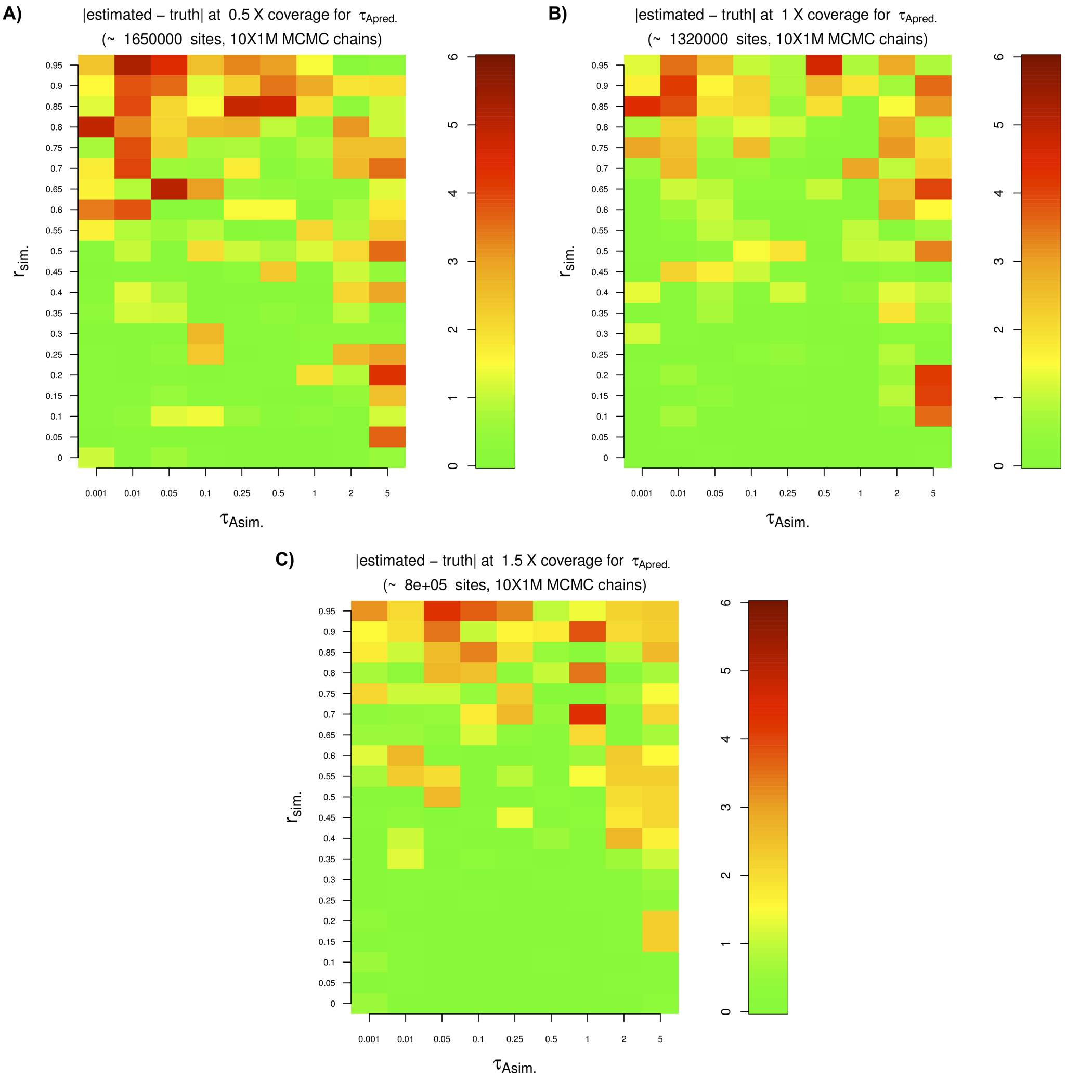

Supplement: S10 Fig — We used a large number of sites and run 10 MCMC chains for 1 million steps each. To ensure convergence, we then selected the chain with the highest posterior probability, and here show estimates from that chain. In all simulations, the anchor drift was set to be equal to the ancient sample drift. A) 0.5X coverage (800,000 simulations). B) 1X coverage (400,000 simulations). C) 1.5X coverage (200,000 simulations). The number of sites with coverage > 0 is denoted at the top of each panel. (TIFF) [file pgen.1005972.s014.tiff]

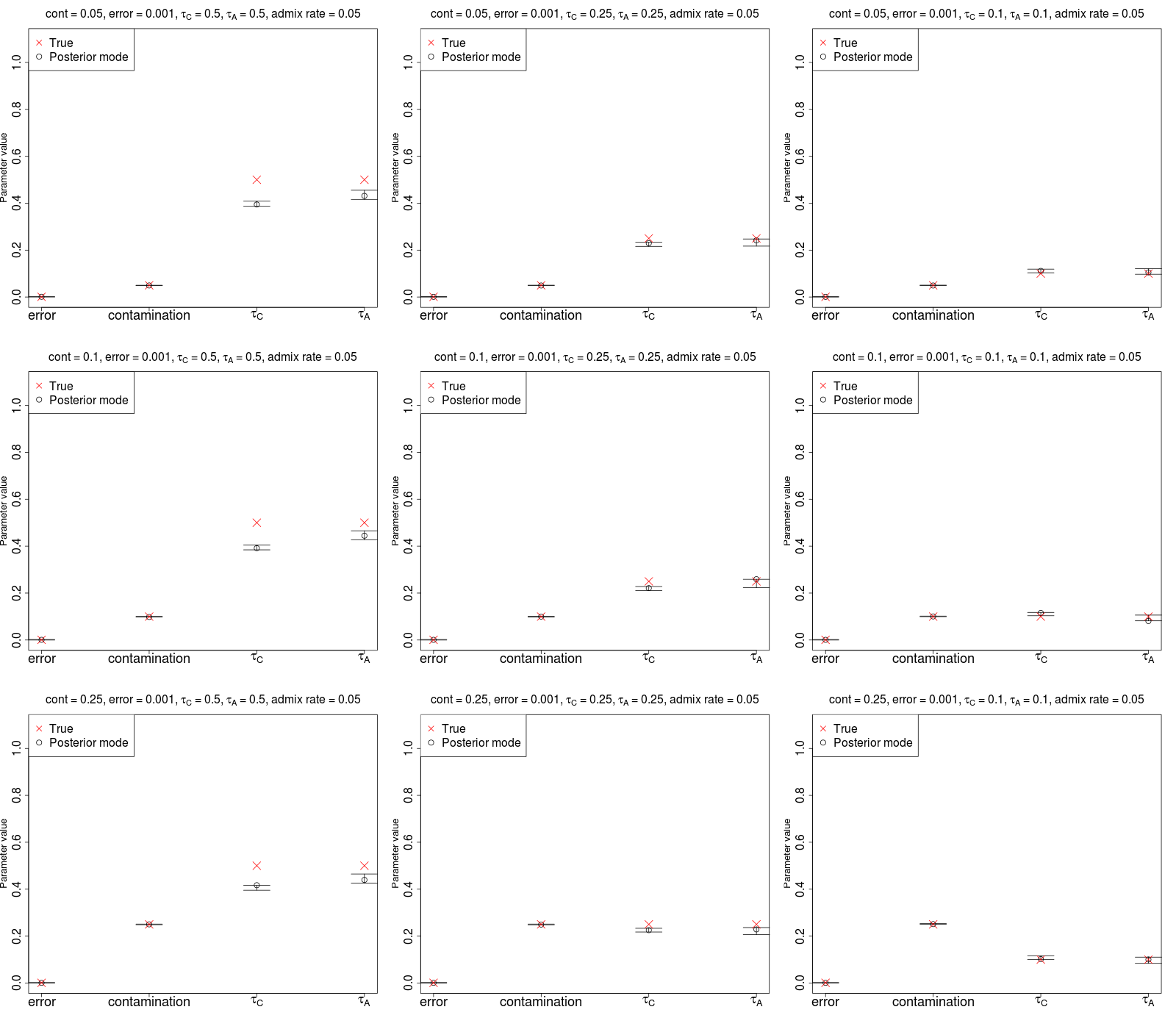

Supplement: S11 Fig — Error bars represent 95% posterior intervals. (TIFF) [file pgen.1005972.s015.tiff]

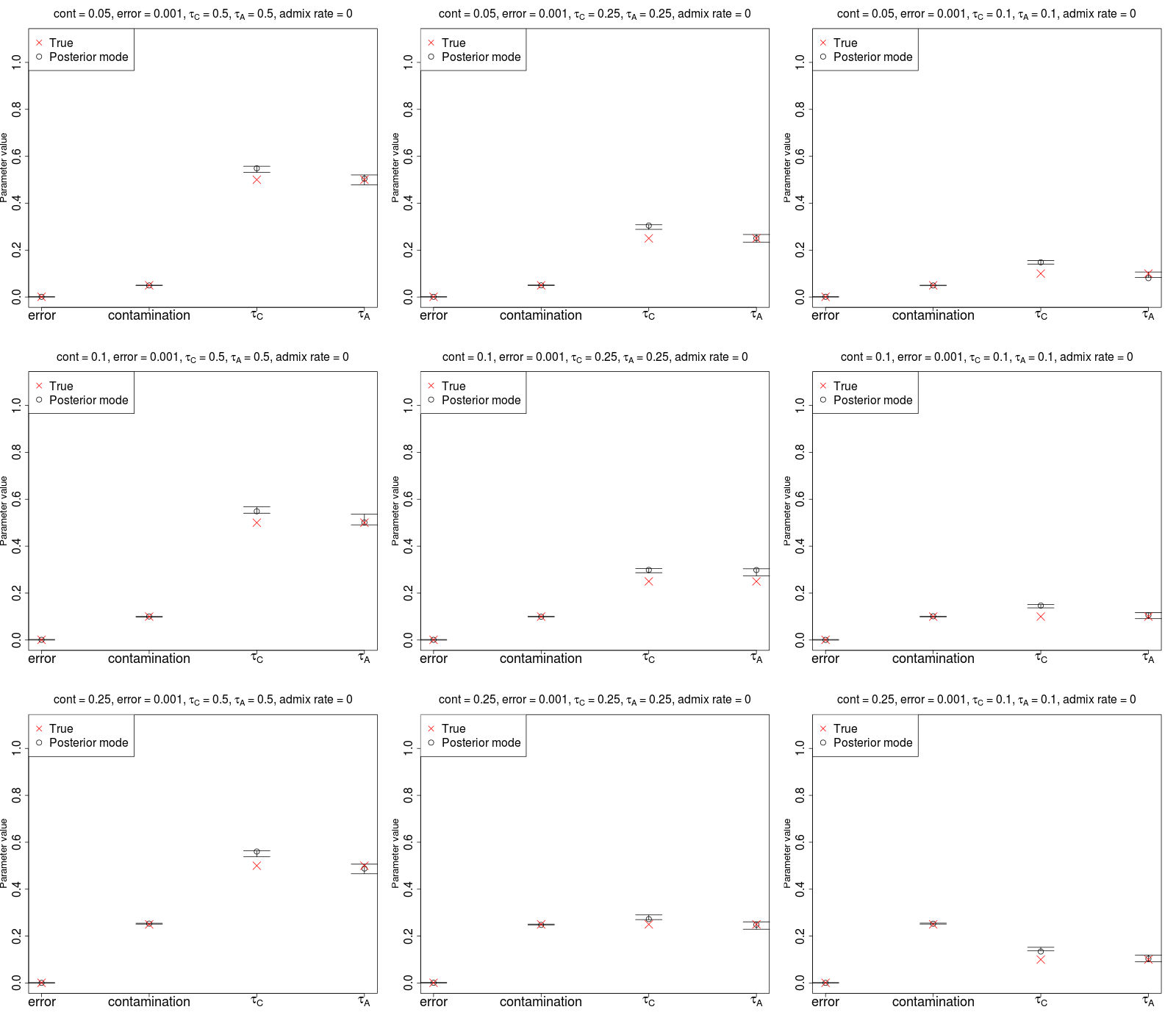

Supplement: S12 Fig — Error bars represent 95% posterior intervals. (TIFF) [file pgen.1005972.s016.tiff]

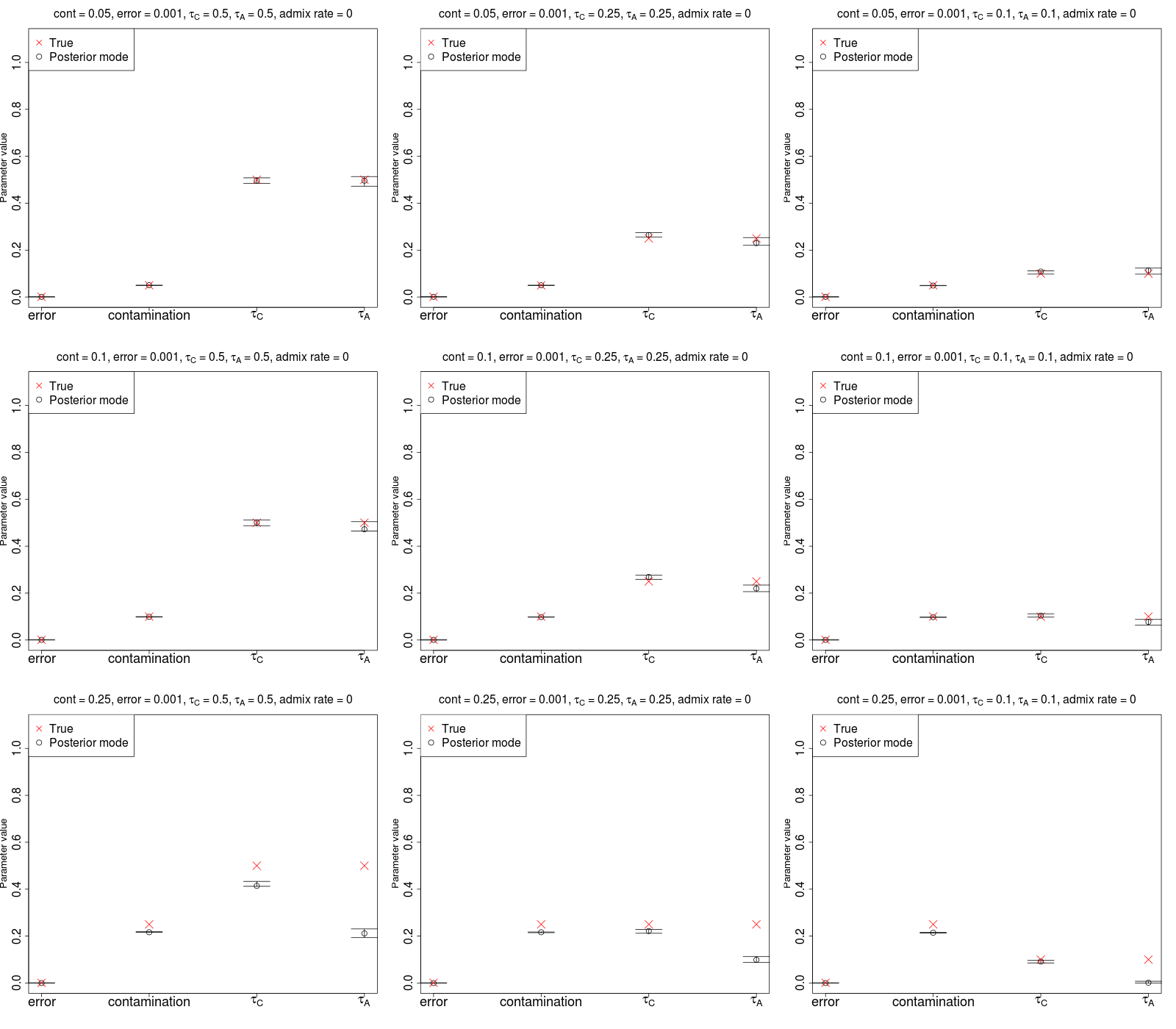

Supplement: S13 Fig — Error bars represent 95% posterior intervals. (TIFF) [file pgen.1005972.s017.tiff]

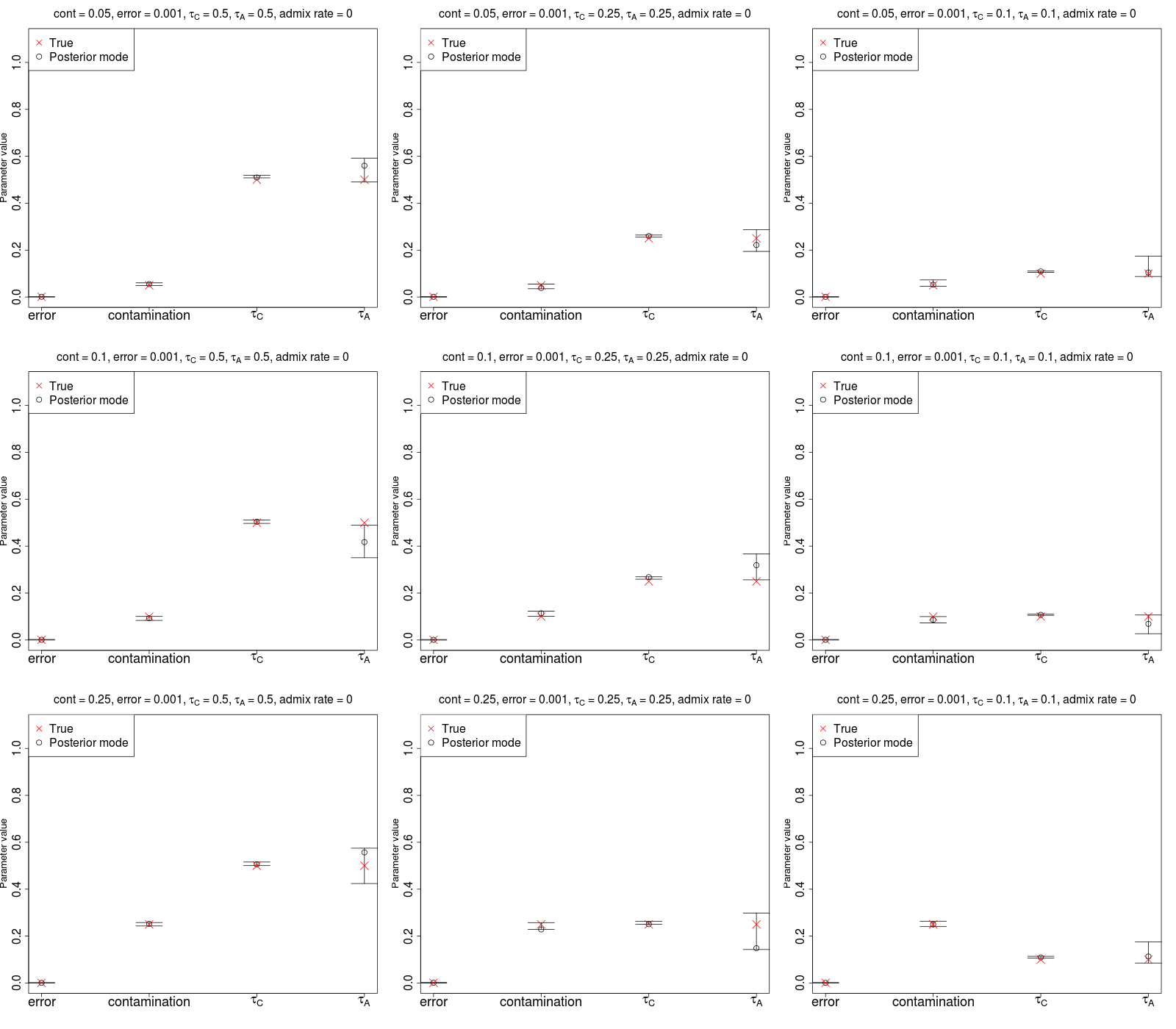

Supplement: S14 Fig — Note that unlike the rest of the simulations, the number of SNPs used in this case was approximately 1.6 million instead of 80,000, and the MCMC chain was run for 1 million steps instead of 100,000. Using a lower number of SNPs or running the chain for a shorter time resulted in inaccurate inferences. Error bars represent 95% posterior intervals. (TIFF) [file pgen.1005972.s018.tiff]

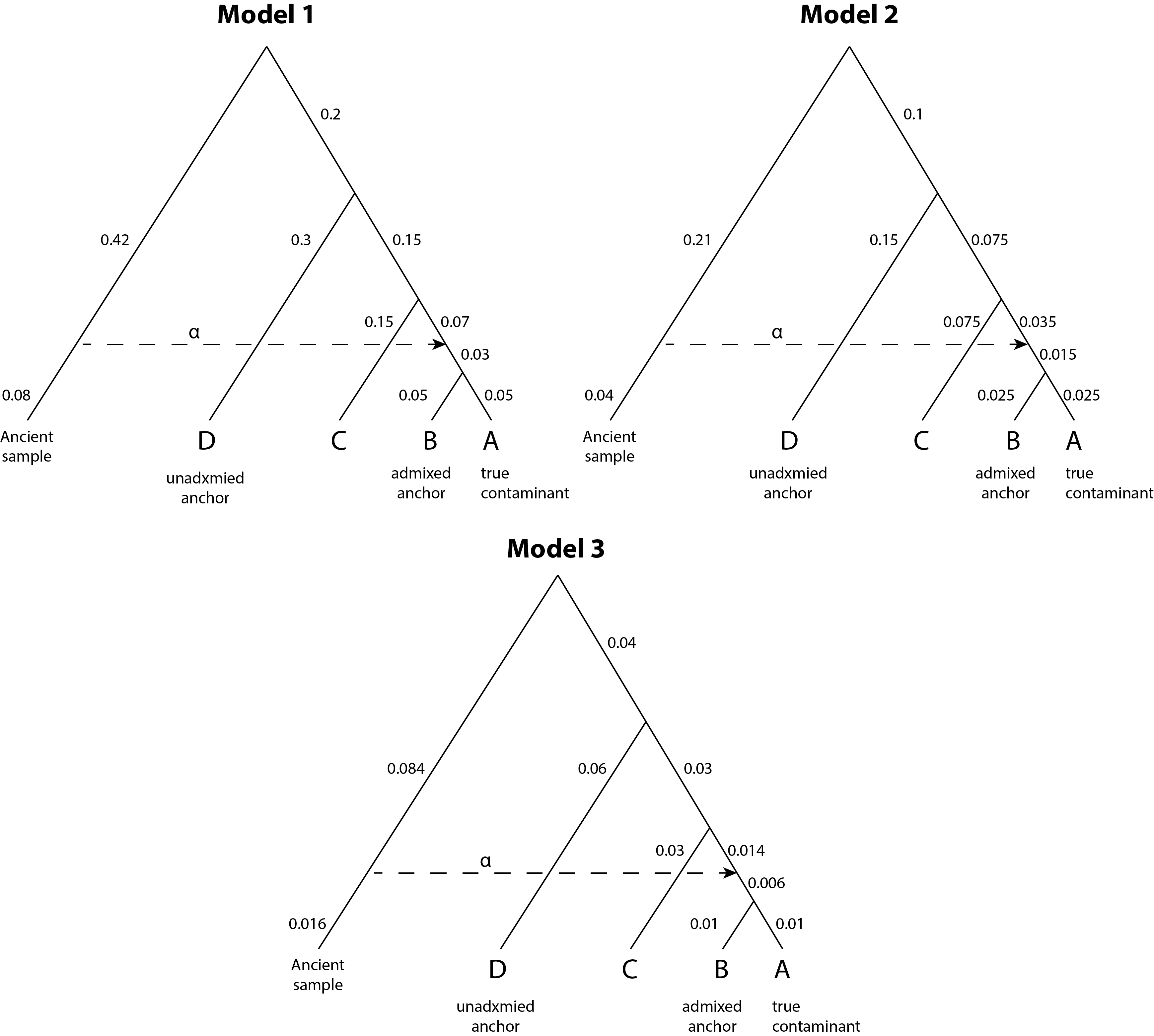

Supplement: S15 Fig — When testing the two-population method, we set panel A as the true contaminant and panel D as the anchor. When testing the three-population method, we set panel A as the true contaminant, panel D as the unadmixed anchor and panel B as the admixed anchor. The numbers on the branches represent the drift parameters. The parameter α represents the admixture rate from the ancient population into the ancestor of A and B. (TIFF) [file pgen.1005972.s019.tiff]

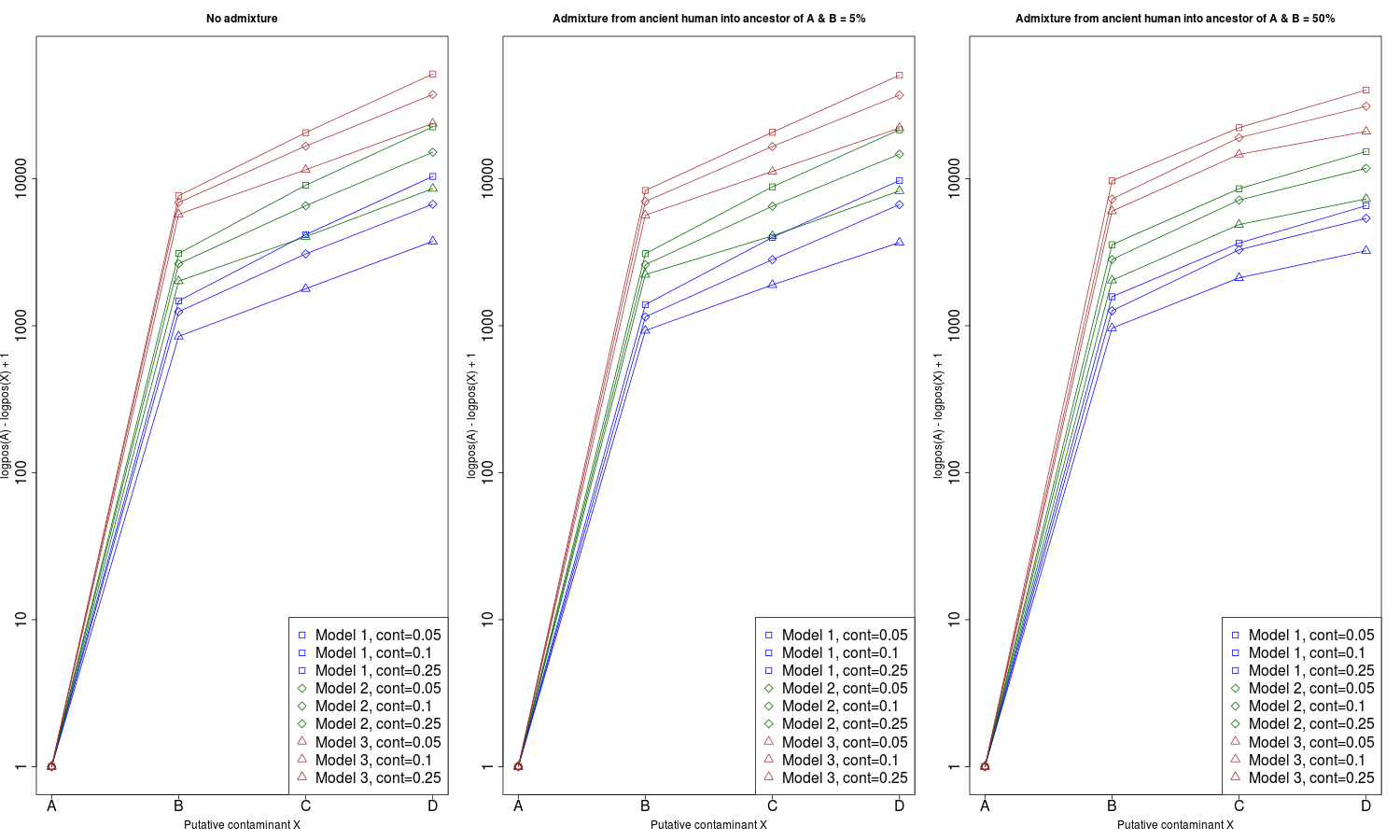

Supplement: S16 Fig — The y-axis shows the difference between the log-posterior for contaminant panel A and the log-posterior for different candidate contaminant panels (A, B, C, D), so low values correspond to high posterior probabilities for each of the candidates. We added a 1 to the difference to be able to plot the difference on a logarithmic scale. The three panels contain results for three admixture scenarios (from left to right: admixture rate of 0%, 5% and 50%) and each panel shows the difference under different contamination rates and demographic models (the population relationships of panels A, B, C and D can be found in S15 Fig). (TIFF) [file pgen.1005972.s020.tiff]

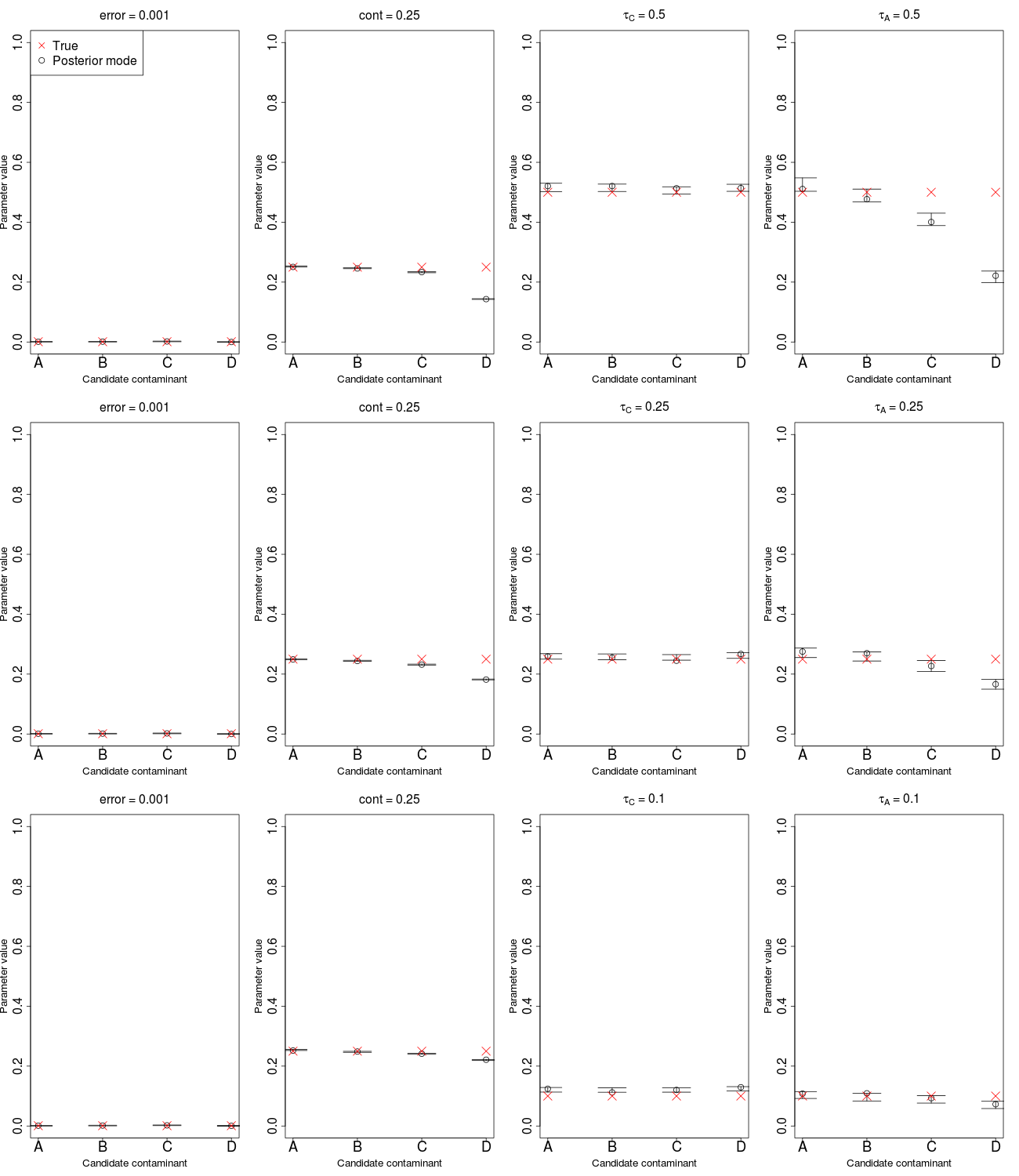

Supplement: S17 Fig — Each row of panels represents a different set of drift parameters, keeping the contamination rate fixed at 25% and the error rate at 0.1%. In this case, the admixture rate from the ancient population to the ancestor of A and B was kept at 0%. The anchor panel used was panel D (the population relationships of panels A, B, C and D can be found in S15 Fig). (TIFF) [file pgen.1005972.s021.tiff]

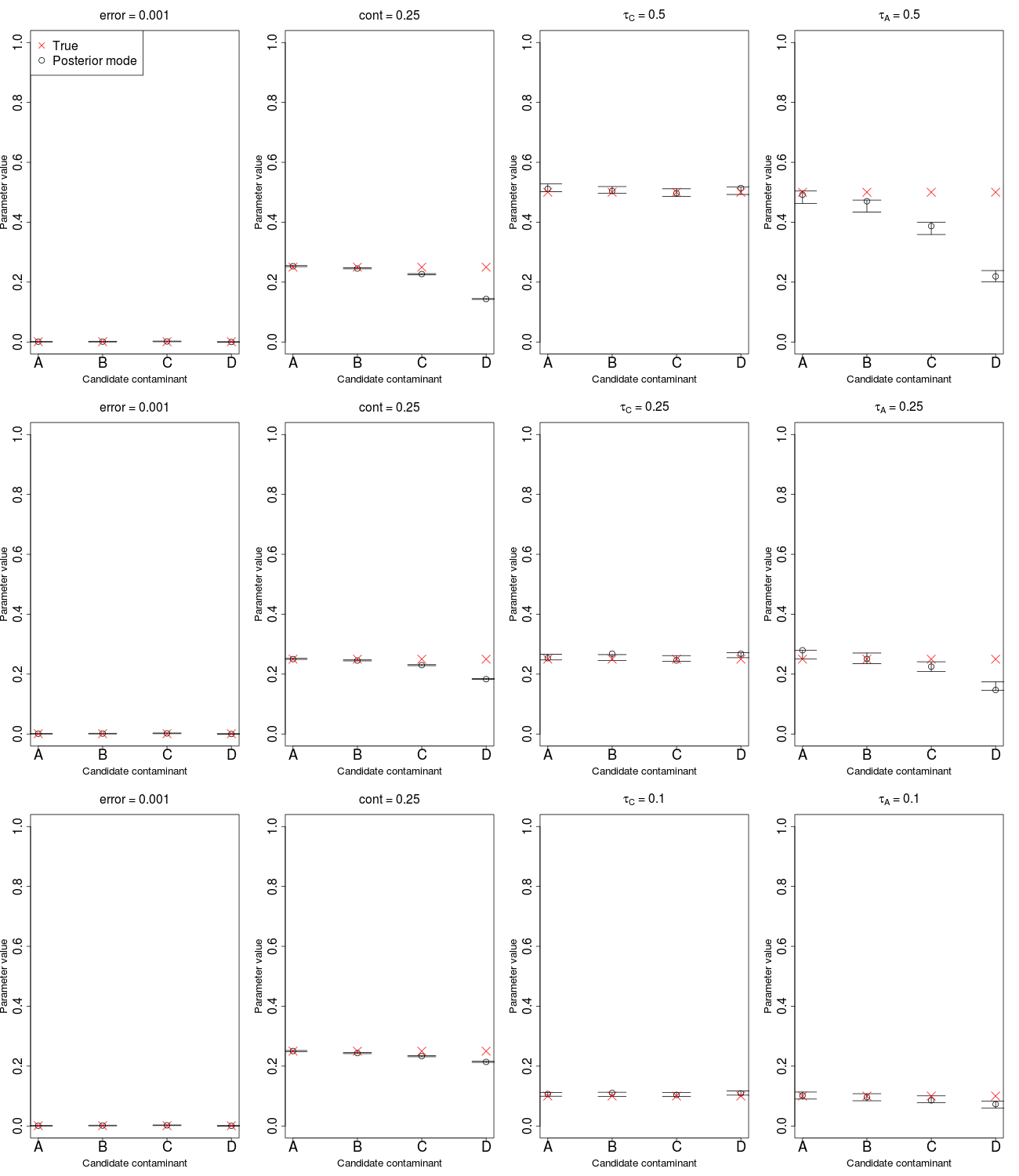

Supplement: S18 Fig — Each row of panels represents a different set of drift parameters, keeping the contamination rate fixed at 25% and the error rate at 0.1%. In this case, the admixture rate from the ancient population to the ancestor of A and B was kept at 5%. The anchor panel used was panel D (the population relationships of panels A, B, C and D can be found in S15 Fig). (TIFF) [file pgen.1005972.s022.tiff]

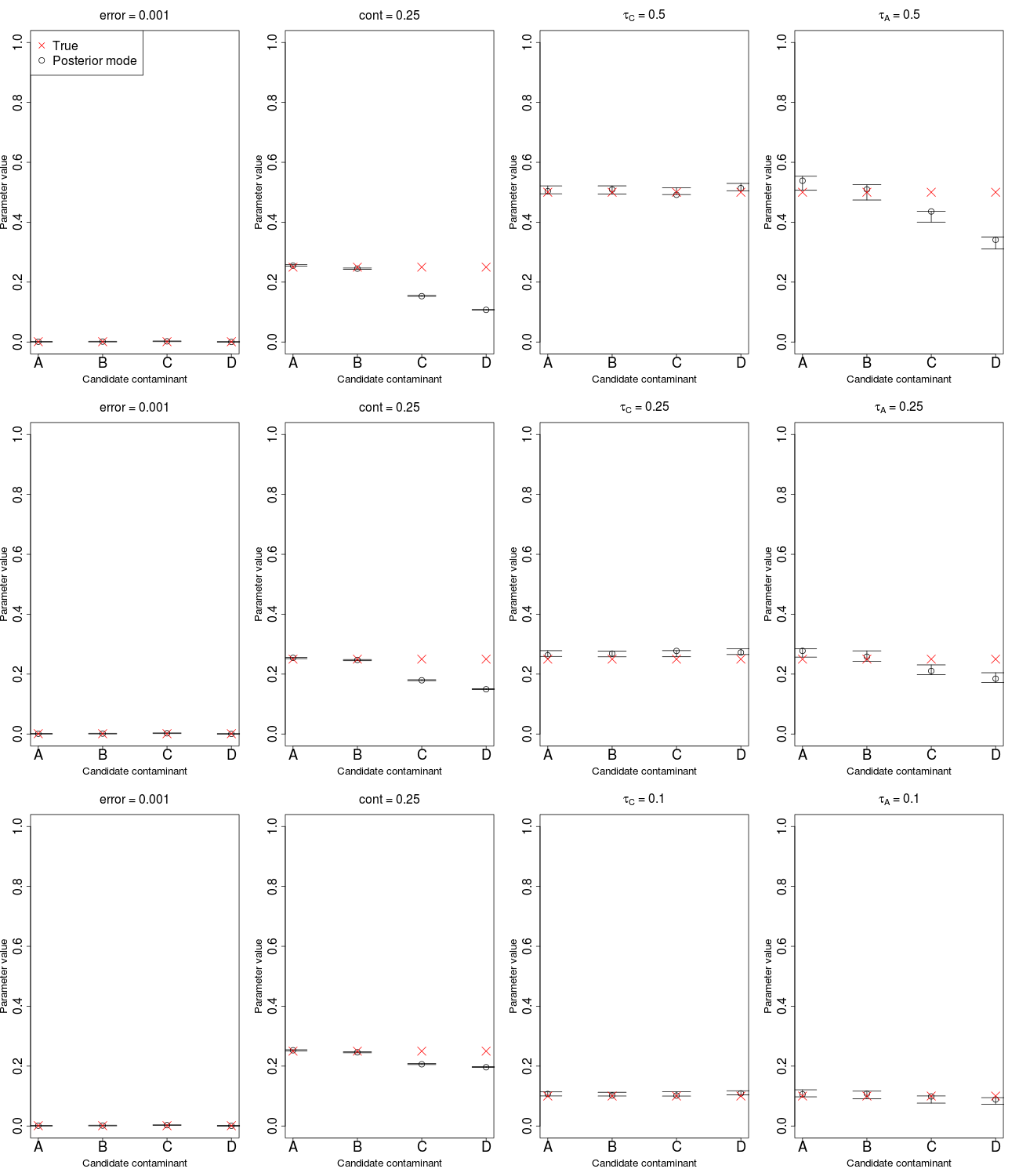

Supplement: S19 Fig — Each row of panels represents a different set of drift parameters, keeping the contamination rate fixed at 25% and the error rate at 0.1%. In this case, the admixture rate from the ancient population to the ancestor of A and B was kept at 50%. The anchor panel used was panel D (the population relationships of panels A, B, C and D can be found in S15 Fig). (TIFF) [file pgen.1005972.s023.tiff]

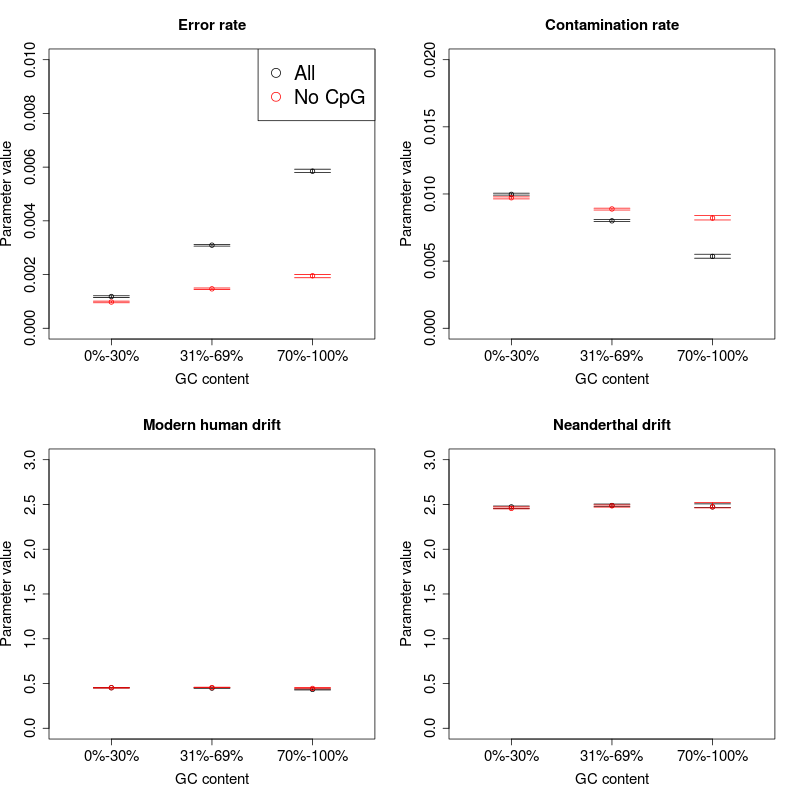

Supplement: S20 Fig — Error bars represent 95% posterior intervals. (TIFF) [file pgen.1005972.s024.tiff]

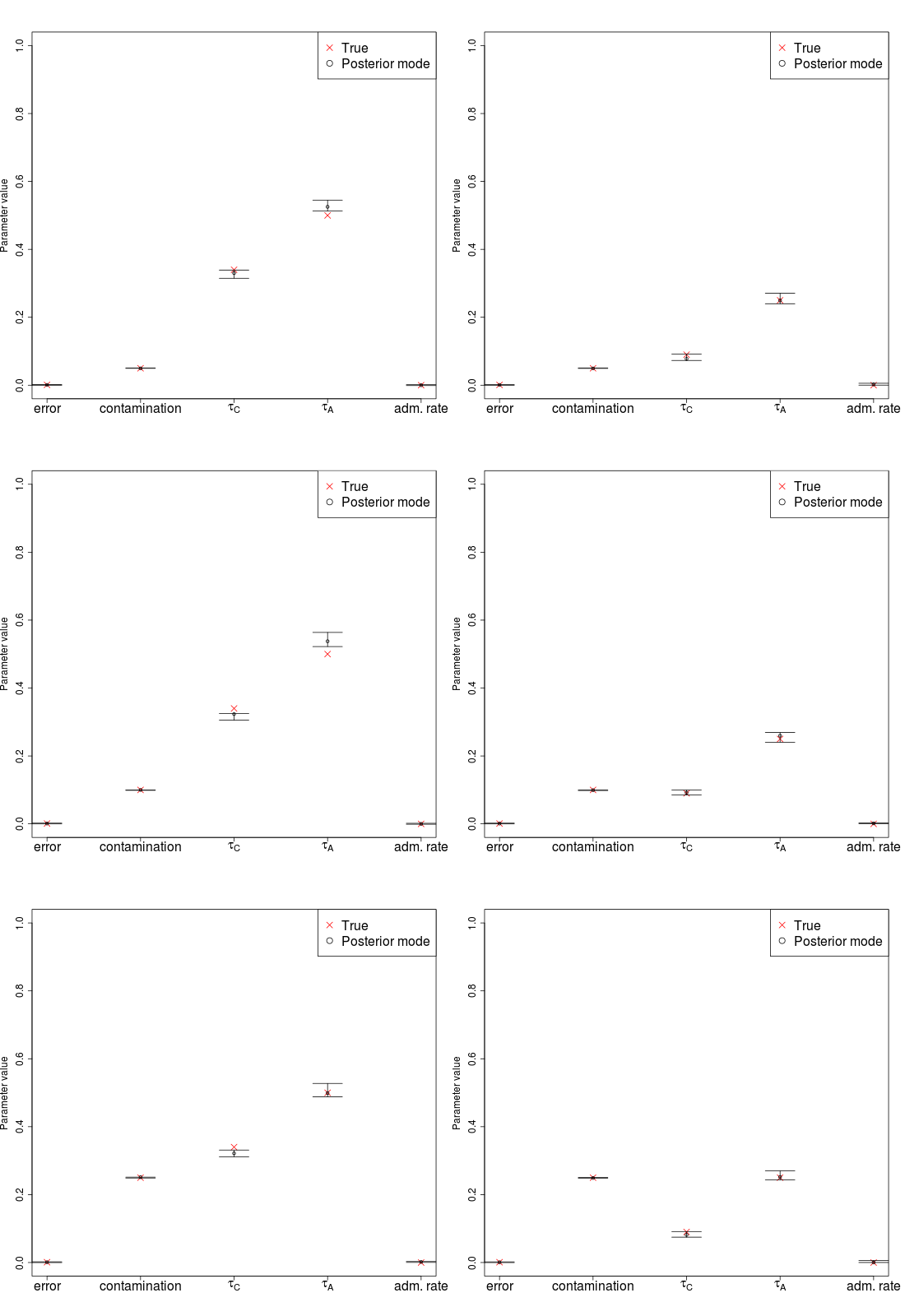

Supplement: S22 Fig — The prior used for the admixture time was uniform over [0.06, 0.1]. Error bars represent 95% posterior intervals. (TIFF) [file pgen.1005972.s026.tiff]

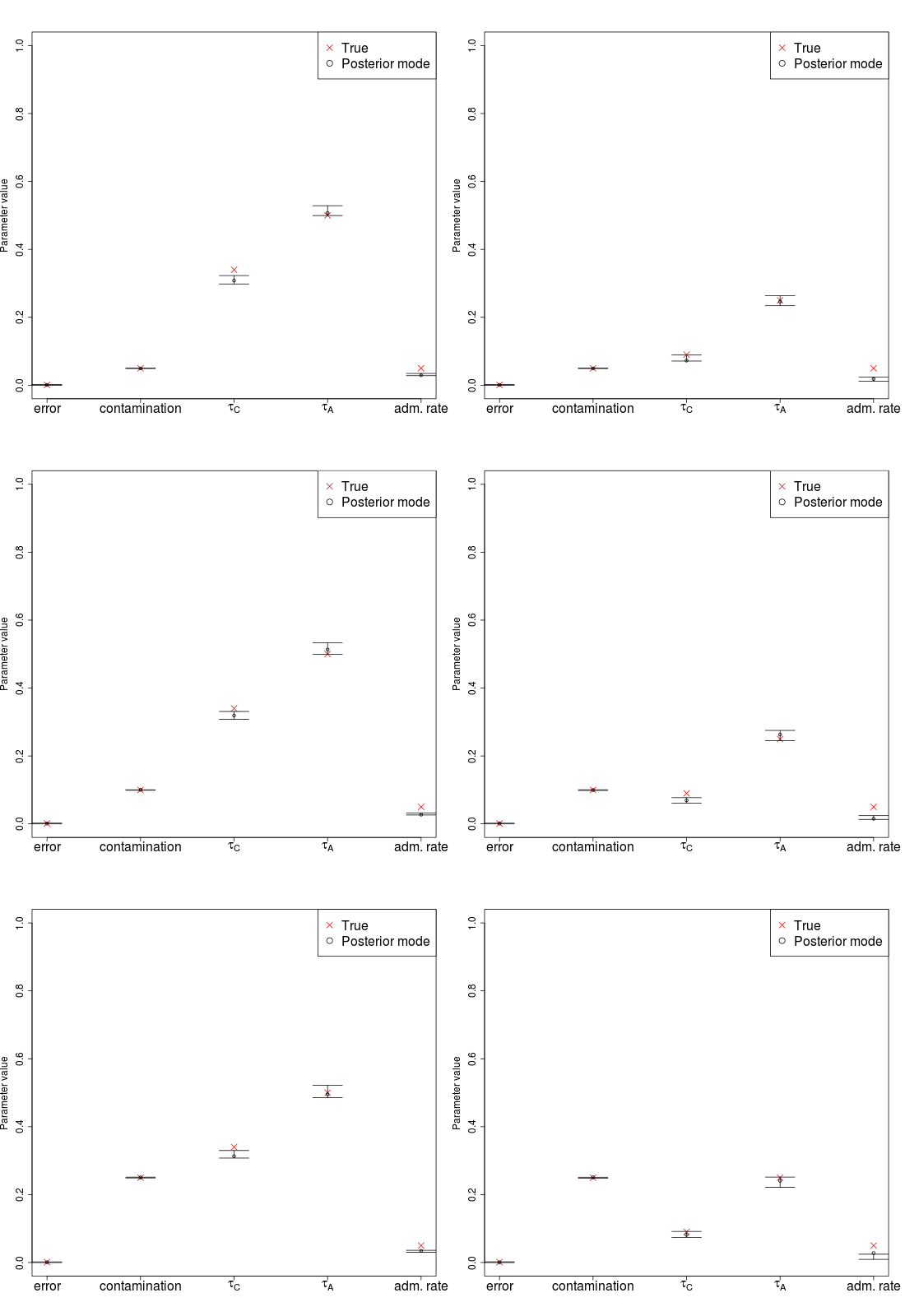

Supplement: S23 Fig — The prior used for the admixture time was uniform over [0.06, 0.1]. Error bars represent 95% posterior intervals. (TIFF) [file pgen.1005972.s027.tiff]

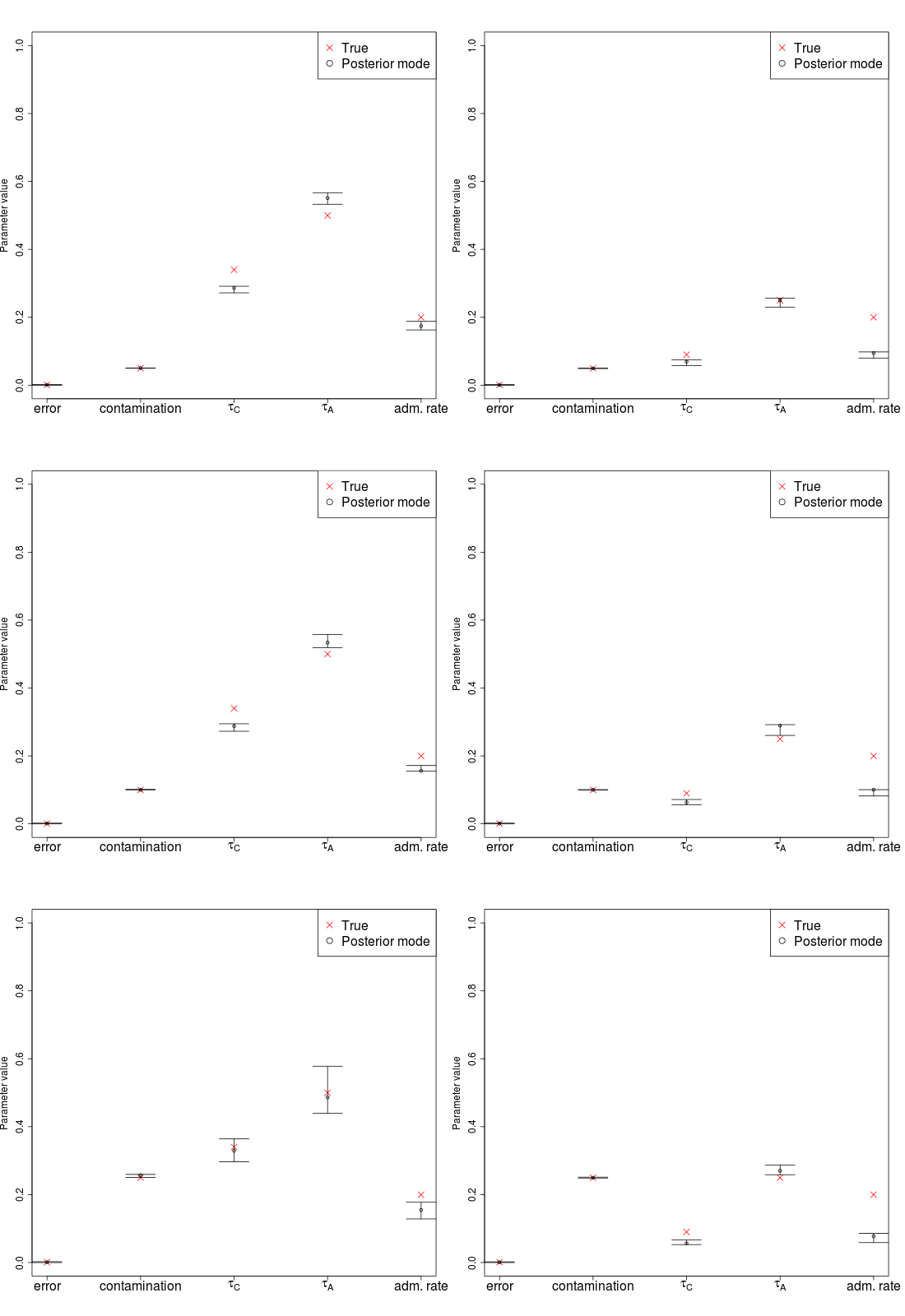

Supplement: S24 Fig — The prior used for the admixture time was uniform over [0.06, 0.1]. Error bars represent 95% posterior intervals. (TIFF) [file pgen.1005972.s028.tiff]

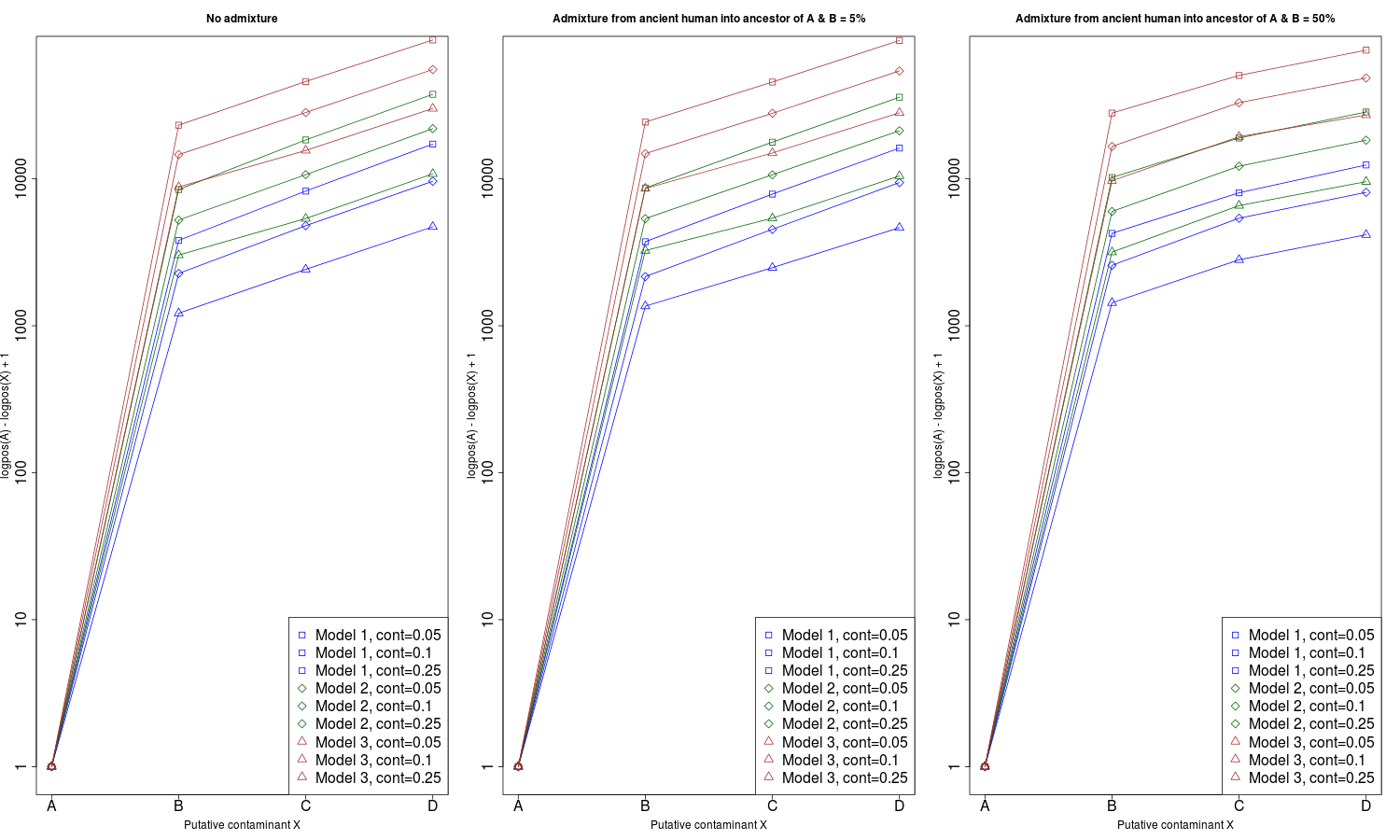

Supplement: S25 Fig — The y-axis shows the difference between the log-posterior for contaminant panel A and the log-posterior for different candidate contaminant panels (A, B, C, D), so low values correspond to high posterior probabilities for each of the candidates. We added a 1 to the difference to be able to plot the difference on a logarithmic scale. The three panels contain results for three admixture scenarios (from left to right: admixture rate of 0%, 5% and 50%) and each panel shows the difference under different contamination rates and demographic models (see S15 Fig). (TIFF) [file pgen.1005972.s029.tiff]

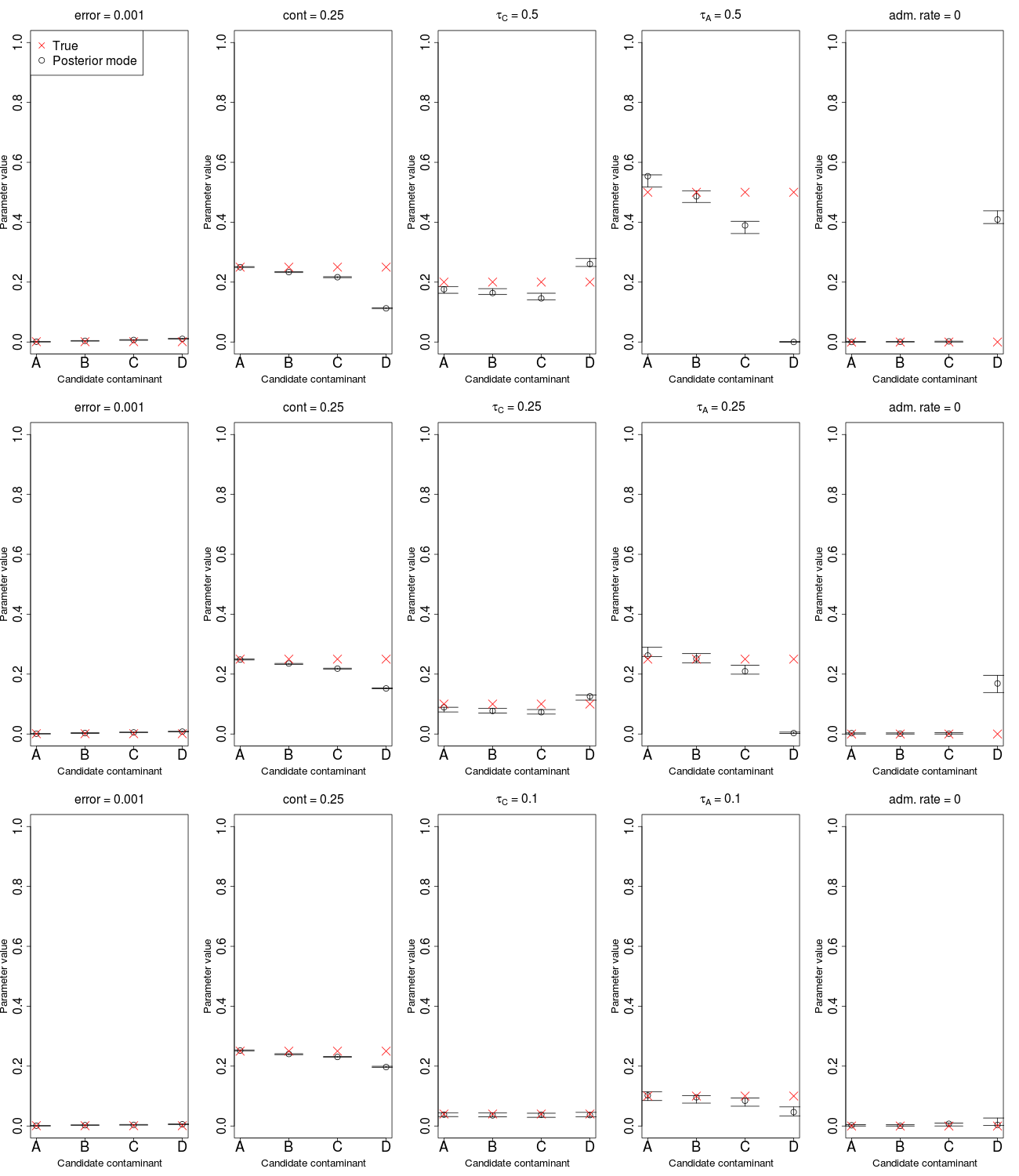

Supplement: S26 Fig — Each row of panels represents a different set of drift parameters, keeping the contamination rate fixed at 25% and the error rate at 0.1%. In this case, the admixture rate from the ancient population to the ancestor of A and B was kept at 0%. The unadmixed anchor panel used was panel D and the admixed anchor panel was panel B (see S15 Fig). (TIFF) [file pgen.1005972.s030.tiff]

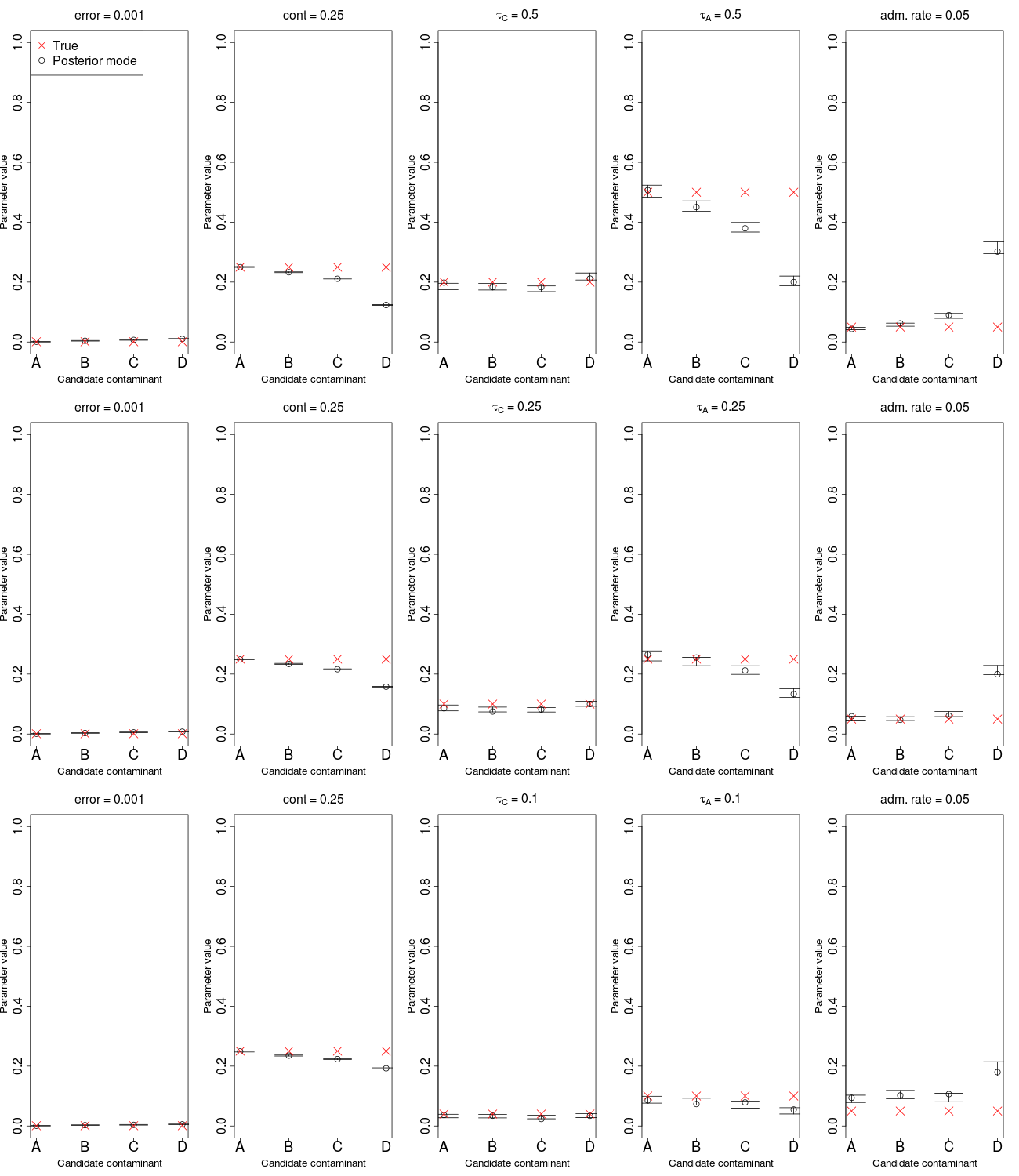

Supplement: S27 Fig — Each row of panels represents a different set of drift parameters, keeping the contamination rate fixed at 25% and the error rate at 0.1%. In this case, the admixture rate from the ancient population to the ancestor of A and B was kept at 5%. The unadmixed anchor panel used was panel D and the admixed anchor panel was panel B (see S15 Fig). (TIFF) [file pgen.1005972.s031.tiff]

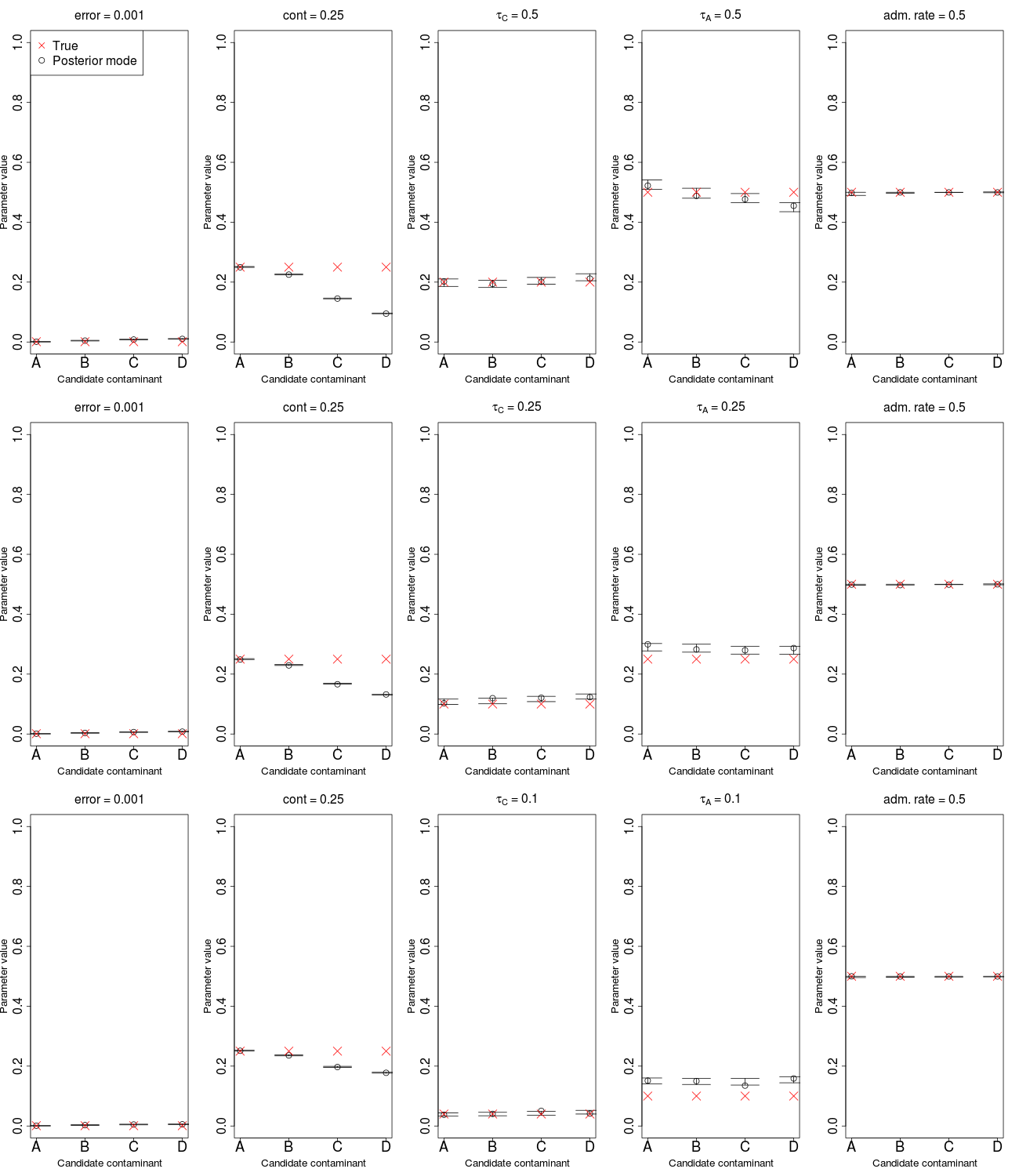

Supplement: S28 Fig — Each row of panels represents a different set of drift parameters, keeping the contamination rate fixed at 25% and the error rate at 0.1%. In this case, the admixture rate from the ancient population to the ancestor of A and B was kept at 50%. The unadmixed anchor panel used was panel D and the admixed anchor panel was panel B (see S15 Fig). (TIFF) [file pgen.1005972.s032.tiff]
