# Supplementary material for: Joint Estimation of Contamination, Error and Demography for Nuclear DNA from Ancient Humans
Source: PLoS Genet. 2016 Apr 6;12(4):e1005972. doi: 10.1371/journal.pgen.1005972 (PMC4822957; doi:10.1371/journal.pgen.1005972)
Supplement: S1 Text — Derivation of formulas to obtain the probabilities of particular genotype states given a demographic history and an anchor population allele frequency, using diffusion theory. (PDF) [file pgen.1005972.s033.pdf]

## S1 Text. Genotype probabilities conditional on a demography

Below we derive formulas 7, 8 and 9 from the main text. Recall that we are interested in calculating the conditional probabilities $P[i|\mathbf{\Omega}, \mathbf{O}] = \mathbf{P}[\mathbf{i}|\mathbf{y}, \tau_\mathbf{C}, \tau_\mathbf{A}]$ for all three possibilities for the genotype in the ancient individual: $i = 0$, 1 or 2. These can be obtained from the definition of conditional probability. Let $f_y^{DD}$ be the joint probability that a site has frequency $y$ ($0 < y < 1$) in the contaminant panel and is homozygous for the derived allele in the ancient individual. Let $f_y^{DA}$ be the joint probability that a site has frequency $y$ in the contaminant panel and is heterozygous in the ancient individual. Finally, let $f_y^{AA}$ be the joint probability that a site has frequency $y$ in the anchor panel and is homozygous for the ancient allele in the ancient individual. Then:

$$P[\ i = 0 \mid y, \tau_C, \tau_A\ ] = \frac{f_y^{AA}}{f_y} = \frac{f_y^{AA}}{f_y^{AA} + f_y^{DA} + f_y^{DD}} \tag{11}$$

$$P[\ i = 1 \mid y, \tau_C, \tau_A\ ] = \frac{f_y^{DA}}{f_y} = \frac{f_y^{DA}}{f_y^{AA} + f_y^{DA} + f_y^{DD}} \tag{12}$$

$$P[\ i = 2 \mid y, \tau_C, \tau_A\ ] = \frac{f_y^{DD}}{f_y} = \frac{f_y^{DD}}{f_y^{AA} + f_y^{DA} + f_y^{DD}} \tag{13}$$

In the above expressions, the functions $f$ depend on $\tau_C$ and $\tau_A$, but we omit this conditioning for ease of notation. As can be seen, all we need to find is the joint probabilities $f_y^{AA}$, $f_y^{DA}$ and $f_y^{DD}$. Here is where diffusion theory comes into play. Let $\phi(y, \tau|x, 0)$ be the Kimura solution to the neutral forward diffusion equation in the absence of mutation [1], given a frequency $x$ at time 0 and an elapsed drift time $\tau$:

$$\phi(y, \tau|x, 0) = 4x(1-x)\sum_{h=1}^{\infty} \frac{2j+1}{j(j+1)} C_{h-1}^{3/2}(1-2x) C_{h-1}^{3/2}(1-2y) e^{-j(j+1)\tau/2} \tag{14}$$

Here, $x$ is the unknown population frequency of the derived allele in the ancestral population and $C_{h-1}^{(3/2)}(\bullet)$ is the Gegenbauer polynomial of order h-1 [2].

Assuming the ancestral population follows an equilibrium frequency distribution $g(x) = \theta/x$, we can write $f_y^{DD}$ as follows:

$$f_y^{DD} = \int_0^1 \phi(y, \tau_C|x, 0) g(x) \left( \int_0^1 z^2 \phi(z, \tau_A|x, 0) dz \right) dx \tag{15}$$

where $z$ is the unknown population frequency of a derived allele in the population to which the ancient individual belongs.

The expression in parentheses is the second moment of the transition density and its solution is known [3]:

$$\int_0^1 z^2 \phi(z, \tau_A|x, 0) dz = x - x(1-x)e^{-\tau_A} \tag{16}$$

This results in:

$$f_y^{DD} = \theta \int_0^1 \phi(y, \tau_C|x, 0)[1 - (1-x)e^{-\tau_A}] dx \tag{17}$$

$$f_y^{DD} = \theta \left[ \int_0^1 \phi(y, \tau_C|x, 0) dx - e^{-\tau_A} \int_0^1 \phi(y, \tau_C|x, 0) dx + e^{-\tau_A} \int_0^1 x \ \phi(y, \tau_C|x, 0) dx \right] \tag{18}$$

The integral of the first two terms of the sum was solved in Chen et al. [4]:

$$\int_0^1 \phi(y, \tau_C|x, 0) dx = e^{-\tau_C} \tag{19}$$

The third term of the sum can be solved by noting that, though the integrand is an infinite sum (i.e. formula 14 multiplied by $x$), only the integrals of the first two terms of that infinite sum are not equal to 0. This can be seen by integrating the parts of the terms of that infinite sum that depend on $x$:

$$\int_0^1 x^2 (1-x) C_{h-1}^{(3/2)} (1-2x) dx = \begin{cases} 1/12 & h = 1 \\ -1/20 & h = 2 \\ 0 & h \geq 3 \end{cases}$$

Therefore, after integrating the first two terms of the infinite sum, we obtain:

$$\int_0^1 x \phi(y, \tau_C|x, 0) dx = \frac{1}{2} e^{-\tau_C} + \left( y - \frac{1}{2} \right) e^{-3\tau_C} \tag{20}$$

So we finally arrive at:

$$f_y^{DD} = \theta\left[e^{-\tau_C} - \frac{1}{2}e^{-\tau_A-\tau_C} + \left(y-\frac{1}{2}\right)e^{-\tau_A-3\tau_C}\right] \tag{21}$$

We can obtain $f_y^{DA}$ in a similar fashion:

$$f_y^{DA} = \int_0^1 \phi(y,\tau_C|x,0)g(x)\left(\int_0^1 2z(1-z)\phi(z,\tau_A|x,0)dz\right)dx \tag{22}$$

Solving the term in the parentheses:

$$\int_0^1 2z(1-z)\phi(z,\tau_A|x,0)dz = 2\left(\int_0^1 z\phi(z,\tau_A|x,0)dz - \int_0^1 z^2\phi(z,\tau_A|x,0)dz\right) \tag{23}$$

The first term of the difference is the first moment of the transition density, which is equal to $x$ [3], while the second term is the second moment (formula 16). Therefore:

$$f_y^{DA} = 2\theta e^{-\tau_A}\left[\int_0^1 \phi(y,\tau_C|x,0)(1-x)dx\right] \tag{24}$$

$$f_y^{DA} = 2\theta e^{-\tau_A}\left[\int_0^1 \phi(y,\tau_C|x,0)dx - \int_0^1 x\ \phi(y,\tau_C|x,0)\ dx\right] \tag{25}$$

And after using formulas 19 and 20, we obtain:

$$f_y^{DA} = \theta\left[e^{-\tau_A-\tau_C} + (1-2y)\,e^{-\tau_A-3\tau_C}\right] \tag{26}$$

To obtain $f_y^{AA}$, we know that, assuming the anchor population to be at equilibrium:

$$f_y = g(y) \tag{27}$$

And therefore:

$$f_y^{AA} + f_y^{DA} + f_y^{DD} = \frac{\theta}{y} \tag{28}$$

So we finally obtain:

$$f_y^{AA} = \theta \left[ \frac{1}{y} - e^{-\tau_C} - \frac{1}{2} e^{-\tau_A - \tau_C} + \left( y - \frac{1}{2} \right) e^{-\tau_A - 3\tau_C} \right] \tag{29}$$

We now have all the elements necessary to obtain the conditional probabilities from formulas 11, 12 and 13, which immediately lead us to formulas 7, 8 and 9.

## References


1. M. Kimura, Solution of a process of random genetic drift with a continuous model, Proceedings of the National Academy of Sciences 41 (1955) 144.

2. M. Abramowitz, I. A. Stegun, Handbook of mathematical functions, Dover New York, 1965.

3. J. F. Crow, M. Kimura, An Introduction to population genetics theory, Harper and Row, New York, Evanston, London, 1970.

4. H. Chen, R. E. Green, S. Pääbo, M. Slatkin, The joint allele-frequency spectrum in closely related species, Genetics 177 (2007) 387–398.
