# Supplementary material for: Joint Estimation of Contamination, Error and Demography for Nuclear DNA from Ancient Humans
Source: PLoS Genet. 2016 Apr 6;12(4):e1005972. doi: 10.1371/journal.pgen.1005972 (PMC4822957; doi:10.1371/journal.pgen.1005972)
Supplement: S2 Text — Explanation of methodology for inferring fragment-specific error parameters in the optional BAM mode of DICE. (PDF) [file pgen.1005972.s034.pdf]

S2 Text. Probabilistic inference using BAM files

Here, we briefly explain the way we infer fragment-specific error parameters in the optional BAM mode of DICE. Let $\mathbb{R}$ be the set of all fragments in the BAM file, and $R_j \in \mathbb{R}$ be a particular aligned fragment of length l. For fragment R_j, let $\{b_{j,1}, ..., b_{j,l}\}$ be the individuals nucleotides in the fragment. At each position of the fragment, there is a specific probability $\kappa_{j,i}$ that the base is erroneous. This probability is provided by the basecaller. Below, we will compute the likelihood of observing a base $b_{j,i} \in R_j$ under a bi-allelic model, given an error rate $\kappa_{j,i}$. Below, we focus on an individual fragment R_j and an individual position i on that fragment, so for simplicity, we drop the subscripts i and j and we let $b_{j,i} = b$ and $\kappa_{j,i} = \kappa$.

Let v be the base that was originally sampled at a given site, before deamination or mismapping. This base could be ancestral or derived. Let $P_{dam}[v \to b]$ be the probability of substitution from v to b due to post-mortem chemical damage. The probabilities of different types of damage (e.g. C→T or G→A) occurring at different positions of a fragment can be computed following Ginolhac et al. [1] and Jónsson et al. [2], producing a matrix that can be provided to DICE as input. We offer the possibility of specifying different post-mortem damage matrices for the endogenous and the contaminant fragments.

Let E denote the event that a sequencing error has occurred, let D the event that chemical damage has occurred, let M be the event that R_j was correctly mapped and let $\neg$ denote the complement of an event (i.e. event has not occurred). We define the probability of observing sequenced base b given that no sequencing error has occurred at a position on a correctly mapped fragment that was originally v, by summing over two possibilities, either chemical damage occurred or it did not:

$$P[b|v, M, \neg E] = \mathbb{1}(v = b) \cdot P[\neg D] + (1 - \mathbb{1}(v = b)) \cdot P[D] \qquad (30)$$

Here, $\mathbb{1}(v = b)$ is an indicator function that is equal to 1 if v is equal to b, and 0 otherwise. The probabilities $P[D]$ and $P[\neg D]$ are respectively equal to $P_{dam}[v \to b]$ and $1 - P_{dam}[v \to b]$.

Subsequently, we compute $P[b|v, M]$, the probability of observing b given v under the assumption that R_j was mapped at the correct genomic location. We have:

$$P[b|v, M] = (1 - \kappa) \cdot P[b|v, M, \neg E] + \kappa \cdot \frac{1}{2} \qquad (31)$$

This is because if a sequencing error has occurred, the probability of observing b is independent of v, and therefore $P[b|v, M, E] = \frac{1}{2}$. Finally, let $P[M]$ be the probability that the fragment R_j is mapped at the correct location as given by the mapping quality. The probability of seeing b given that v was the base that was sampled before deamination is then:

$$P[b|v] = P[M] \cdot P[b|v, M] + P[\neg M] \cdot \frac{1}{2} \tag{32}$$

The probability of observing b given that the fragment was mismapped is independent of v, hence $P[b|v, \neg M] = \frac{1}{2}$. If either the base quality or mapping quality indicate a probability of error of 100%, $P[b|v]$ will be equal to $\frac{1}{2}$. These probabilities are used instead of the genome-wide error term ϵ in equations **??**, **??** and **??**. For instance, equation **??** for a specific base b in fragment R_j becomes:

$$\begin{aligned} q_2 = r_C(w \cdot P[b = der|v = der,\ contaminant]+ \\ (1-w) \cdot P[b = der|v = anc,\ contaminant])+ \\ (1-r_C) \cdot P[b = der|v = der,\ ancient] \end{aligned} \tag{33}$$

Here, *der* is the derived base and *anc* is the ancestral base. In case different post-mortem damage matrices are provided by the user for the ancient and the contaminant fragments, the events *contaminant* and *ancient* serve to denote which damage probabilities (i.e. P_{dam}) should be used in each case.

References

1. A. Ginolhac, M. Rasmussen, M. T. P. Gilbert, E. Willerslev, L. Orlando, mapdamage: testing for damage patterns in ancient dna sequences, Bioinformatics 27 (2011) 2153–2155.

2. H. Jónsson, A. Ginolhac, M. Schubert, P. L. Johnson, L. Orlando, mapdamage2.0: fast approximate bayesian estimates of ancient dna damage parameters, Bioinformatics 29 (2013) 1682–1684.
